# Multiple Co-Evolutionary Networks Are Supported by the Common Tertiary Scaffold of the LacI/GalR Proteins

**DOI:** 10.1371/journal.pone.0084398

**Published:** 2013-12-31

**Authors:** Daniel J. Parente, Liskin Swint-Kruse

**Affiliations:** Department of Biochemistry and Molecular Biology, The University of Kansas Medical Center, Kansas City, Kansas, United States of America; University of Edinburgh, United Kingdom

## Abstract

Protein families might evolve paralogous functions on their common tertiary scaffold in two ways. First, the locations of functionally-important sites might be “hard-wired” into the structure, with novel functions evolved by altering the amino acid (e.g. Ala vs Ser) at these positions. Alternatively, the tertiary scaffold might be adaptable, accommodating a unique set of functionally important sites for each paralogous function. To discriminate between these possibilities, we compared the set of functionally important sites in the six largest paralogous subfamilies of the LacI/GalR transcription repressor family. LacI/GalR paralogs share a common tertiary structure, but have low sequence identity (≤30%), and regulate a variety of metabolic processes. Functionally important positions were identified by conservation and co-evolutionary sequence analyses. Results showed that conserved positions use a mixture of the “hard-wired” and “accommodating” scaffold frameworks, but that the co-evolution networks were highly dissimilar between any pair of subfamilies. Therefore, the tertiary structure can accommodate multiple networks of functionally important positions. This possibility should be included when designing and interpreting sequence analyses of other protein families. Software implementing conservation and co-evolution analyses is available at https://sourceforge.net/projects/coevolutils/.

## Introduction

The study of protein evolution has been energized by the genomic revolution, which greatly expanded the numbers of sequences that associate into various protein families. Within families, proteins have similar tertiary structures and functional similarities, which can occur with as low as 15% sequence identity. Clusters of higher sequence identity delimit protein subfamilies, which usually exhibit even greater structural/functional similarity. In fact, homologs within a subfamily can be orthologous to each other; that is, they perform the same functional role in different organisms. Between subfamilies, the functional relationship can be paralogous; that is, members of different subfamilies perform distinct activities. For example, paralogous repressor proteins may control different genes within the same organism.

For a given tertiary scaffold, the evolution of paralogous and orthologous functions might be accomplished by two routes: First, the scaffold might be “hard-wired” with key functional locations; changing the amino acids at these positions would alter function. Second, the scaffold might be adaptive: the locations of key functional positions could move on an “accommodating” tertiary structure. (In intrinsically disordered regions of proteins, functional variation can also arise from the acquisition of novel structure or from rapid evolution [1].) We have explored these two possibilities using subfamilies in the LacI/GalR transcription regulators as a model system. The LacI/GalR family is experimentally well-characterized for both wild-type (reviewed in [2], [3]) and synthetic homologs [4]–[6] and is often used to develop and evaluate bioinformatics methods [7]–[15]. Here, our strategy was to (i) use evolutionary information to identify functionally important positions that occur within LacI/GalR subfamilies and then (ii) compare the locations of these positions between paralogous subfamilies.

**Figure 1:**
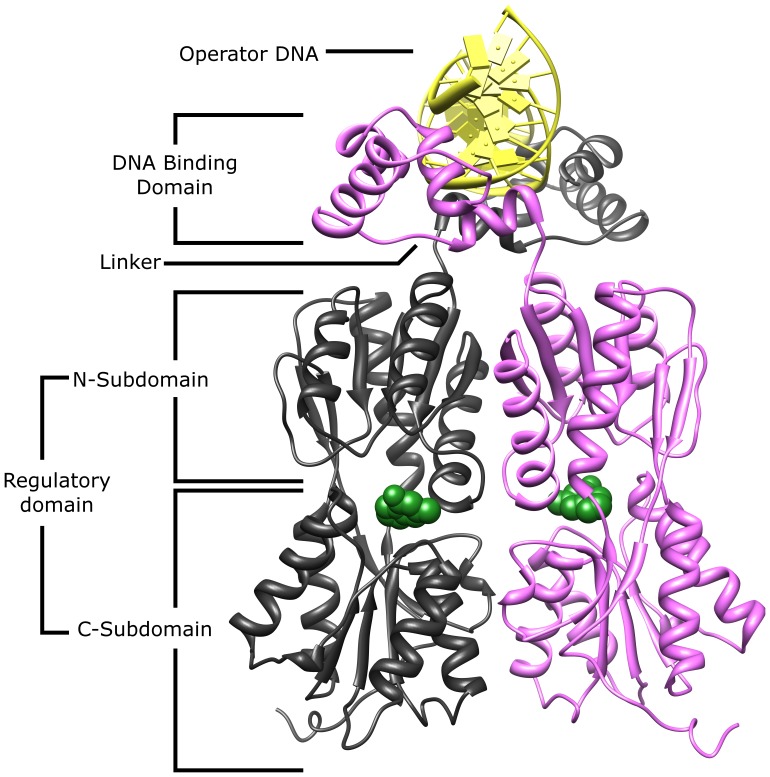
Representative structure of a LacI/GalR protein. The homodimeric functional units of LacI/GalR repressors comprise an N-terminal DNA binding domain connected to a C-terminal regulatory domain by an 18-amino acid linker. The regulatory domain is further subdivided into N- and C-subdomains. The core function of LacI/GalR proteins is to control gene expression in response to the presence of various allosteric effectors. In most LacI/GalR proteins, these effectors (green, spacefilled) bind at the cleft between the N- and C-regulatory subdomains which triggers a conformational change that substantially alters DNA binding. This causes induction (diminished DNA binding) for most members of the LacI/GalR family. For PurR and CcpA, endogenous effector binding enhances DNA binding (Table 1). Further, CcpA is primarily allosterically regulated by a protein-protein interaction with HPr-Ser46-P, with only a secondary, ‘fine-tuning’ role for small-molecule effectors [87]. The structure shown here is the *E. coli* lactose repressor (PDB: 1efa) [18]. Some members of the family also have known accessory functions, such as the ability to undergo tetramerization to accomplish DNA looping; LacI accomplishes this by an C-terminal helical tetramization domain (not shown), while GalR tetramerizes along the surface of the regulatory C-subdomain [63].

The LacI/GalR family contains >2000 homologs that cluster by sequence identity into 45 subfamilies [14] (and unpublished data). All characterized family members bind operator DNA sites to regulate gene transcription; many are allosterically modulated by binding small molecules. Representatives from a variety of subfamilies regulate different aspects of bacterial metabolism [2], [14], [16]. Fourteen known *E. coli* paralogs fall into different subfamilies, whereas two *E. coli* isorepressors (GalR and GalS) [17] fall into a fifteenth subfamily [14]. Representative crystal structures from 17 subfamilies show similar tertiary structures (Figure 1) [2], [18]–[32]. For example, structure alignments of the *E. coli* paralogs LacI and PurR shows a 1.75 Å RMSD, despite 26% sequence identity [18], [21], [33]. These two proteins respectively bind the *lacO* and *pur* operators [2], [3], [34]–[36], and are respectively regulated by binding allolactose and purines [3], [37]–[39]. Thus, the LacI/GalR sequence clusters appear to correspond to groups of orthologs, with each subfamily paralogous to the others.

**Figure 2:**
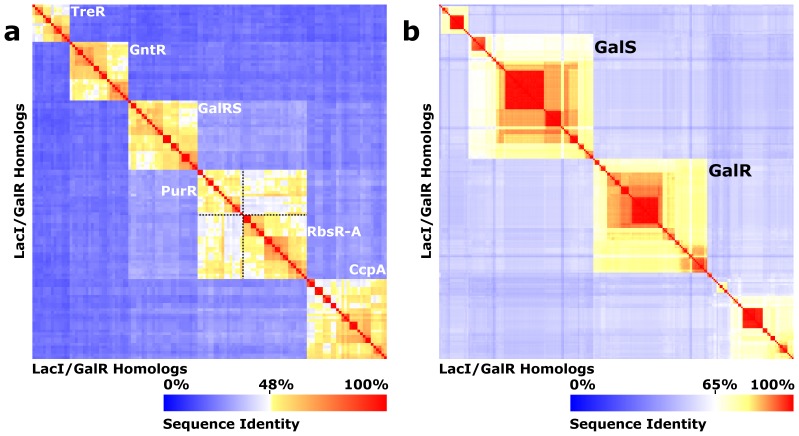
LacI/GalR subfamily sequence clustering. All-vs-all sequence identity heatmaps [14] are shown for (a) the CcpA, GalRS, GntR, PurR, RbsR-A, and TreR subfamilies, and (b) the GalRS subfamily. The X and Y axes correspond to representative homologs drawn from the indicated subfamilies. Sequence identity is shown according to the color scale at the bottom of each panel. Note that the heatmap color gradient differs between panels (a) and (b). The sequences in panel (a) cluster into six sequence identity groups (orange boxes) with clear discontinuities between them. Sequence identity between most of the *Escherichia coli* paralogs was ≤30%, although the RbsR-A and PurR subfamilies had a higher sequence identity relationship (45% between *E. coli* paralogs), and their threshold was less distinct; to aid visual inspection, the boundaries are shown with dotted black lines. (b) The GalRS subfamily contains 5 subclusters. The two *E*. *Coli* isorepressors, GalR and GalS fall into two of these.

Phylogenetic analysis suggests that the LacI/GalR subfamilies diverged after a single event, in which a periplasmic binding protein acquired a DNA-binding domain and the ability to form homodimer [40]. Here, we used the 6 largest subfamilies – CcpA, GalRS, GntR, PurR, RbsR-A, and TreR – (Table 1 and Figure 2a) to compare and contrast the amino acid positions on the common tertiary structure that are under evolutionary constraint. (*E. coli* LacI, the oldest member of this family and commonly-used to provide a reference numbering system, had fewer than 100 sequences in its subfamily and therefore was not included in the present analysis.) One signal for evolutionary constraint was sequence conservation. A second signal was co-evolution, which can be detected when pairs of amino acid positions vary together during evolution. That is, if one position in a co-evolving pair is mutated, its partner usually has a corresponding secondary mutation. Importantly, the second mutation is not random; pairs of amino acids preferentially occur together during evolution. Co-evolving positions have been proposed to be involved in structural contact [41]–[46], allosteric communication [47]–[49], conformational change [50] and thermodynamic coupling [51], [52].

**Table 1:** Key parameters for the LacI/GalR subfamilies.

Subfamily	Num. Sequences	Internal identity	Regulatory function	Allosteric effector(s)	Allosteric effect
GntR [90], [91]	159	39–99%	Gluconate utilization	Gluconate, Gluconate-6-P	Induction
TreR [92]	115	35–99%	Trehalose utilization	Trehalose-6-P, Trehalose	Induction, Anti-induction
CcpA [87], [93]–[96]	211	39–99%	Gram (+) carbon metabolism	HPr-Ser46-P, Crh-Ser46-P, Glucose 6-P, Fructose-1,6-P, NADP	Co-repression
GalRS [17], [97]	230	44–99%	Galactose utilization	Galactose, Fucose	Induction
» GalR	74	62–99%			
» GalS	81	57–99%			
PurR [98]–[100]	160	36–99%	Purine metabolism	Guanine, Hypoxanthine	Co-repression
RbsR-A [101]–[103]	180	40–99%	Ribose metabolism	D-Ribose	Induction

A variety of algorithms have been developed to detect co-evolution [43], [44], [47], [51], [53]–[56]. Each method uses a multiple sequence alignment (MSA) to calculate scores that describe the strength of evolutionary constraint between each pair of amino acid positions. However, different algorithms rank different pairs as the most strongly co-evolving [54], with no single algorithm clearly more “correct” than others. Thus, for this work, we used five common methods – ZNMI, OMES, McBASC, ELSC and SCA – that employ divergent strategies to detect evolutionary constraints. ZNMI uses information theory [53]; OMES calculates a goodness-of-fit-like statistical parameter [47], [54]; McBASC detects coordinated changes in amino acid similarity [44], [55], [56]; and ELSC [43] and SCA [51] use a statistical perturbation approach. As expected, when the LacI/GalR subfamilies were analyzed, each co-evolution algorithm returned different outputs. Nevertheless, when results were compared *between* subfamilies, all algorithms supported the same conclusions. Thus, unless a specific algorithm is named, results were pertinent to all five of the co-evolution analyses.

## Results

### Subfamily-specific Conservation is Widespread among the LacI/GalR Subfamilies

**Figure 3:**
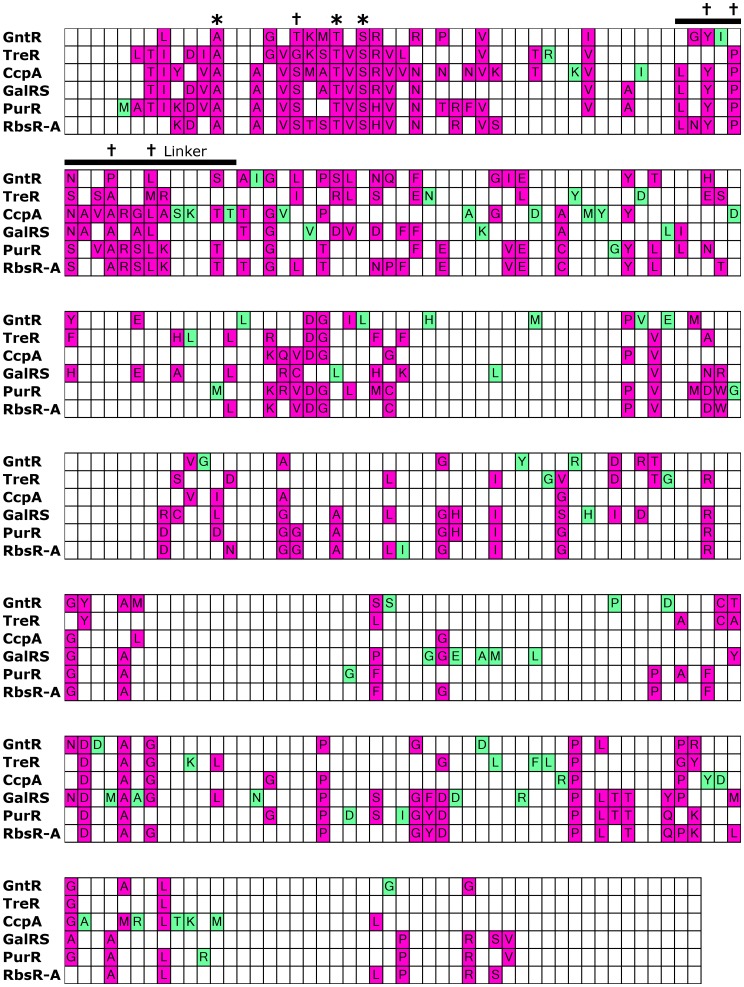
Comparison of conserved sites among subfamilies. The alignment comprising reference sequences for each of the subfamilies was color coded to show sites of conservation. The linker sequence (black bar) is highlighted to show the separation of the N-terminal DNA binding domain from the C-terminal regulatory domain. Colored cells denote that a position is conserved *within* a given, individual subfamily (<5% sequence variability, consistent with our definition of conservation during co-evolution analysis, see Methods); white cells denote non-conserved positions within a subfamily or gaps in the reference sequence. The amino acid shown within each conserved cell is the consensus amino acid. Positions that are conserved in only one of the six subfamilies are highlighted in green, while those conserved in multiple subfamilies (two or more) are highlighted in magenta. In an alignment of representative sequences drawn from 34 LacI/GalR subfamilies [14], only three positions (asterisks; 10, 19 and 21) were conserved (as defined by 5% sequence variability). Only 5 additional positions were conserved (daggers; 16, 47, 49, 53 and 56) among more closely-related subfamilies that contain a functionally-important “YxPxxxAxxL” (YPAL) motif in the linker region [14].

Within a protein family or subfamily, conserved positions are identified based on their low sequence entropies [57]. In this work, we first assessed whether the locations of conserved positions were constant between the six paralogous subfamilies. Along diverging lineages, varied conservation has been referred to as “heterotachy” or “class I functional divergence” [58]–[61]. To that end, we calculated the sequence entropy for each position within a subfamily and projected the values onto a reference sequence. The reference sequences for the six subfamilies were then aligned and the locations of conserved positions *within* each subfamily were compared. (Figure 3; note that sequence alignments were benchmarked against structural alignments for the available crystal structures of CcpA, PurR, and TreR, see Methods). Results revealed locations of both similarity and dissimilarity. About 19% of all positions (63 of ∼332) were conserved among at least four of the six subfamilies (Figure 3, magenta). Many of these positions localized to the core of the DNA binding and regulatory subdomains and to binding surfaces of the LacI/GalR tertiary structure. The common locations of the conserved positions are consistent with a “hard-wired” component of the tertiary scaffold. In contrast, 72 positions were conserved in only one of the six subfamilies (Figure 3, green). On average, each subfamily had 12 uniquely-conserved positions, which must be crucial for function of the specific subfamily. The number of uniquely-conserved positions varied considerably among subfamilies. RbsR-A and PurR had the fewest number of uniquely conserved positions, which might be a result of their greater sequence relatedness to each other (Figure 2). CcpA and GalRS both had many uniquely-conserved positions, probably as a result of the need to conserve their unique protein-protein interaction surfaces. Notably, none of these positions would be identified by analyses that identify evolutionary patterns that persist across the entire LacI/GalR family.

One limitation of this analysis is that increasing the number of subfamilies could reveal that “uniquely” conserved sites are common to additional subfamilies. However, the fact that each of the 6 subfamilies frequently failed to conserve positions that were conserved in other subfamilies suggested that various features of the tertiary structure are either redundant or adaptive. Further, the differences were reflected in the conservation of the whole family: Surprisingly, only three positions were conserved among the whole family (Figure 3, asterisks); only 8 positions were conserved in a major functionally-important subgroup that comprises 22 of the 34 subfamilies originally described, including 5 of the 6 in this study (Figure 3, * and †) [14]. These low numbers are consistent with widespread, subfamily-specific conservation.

**Figure 4:**
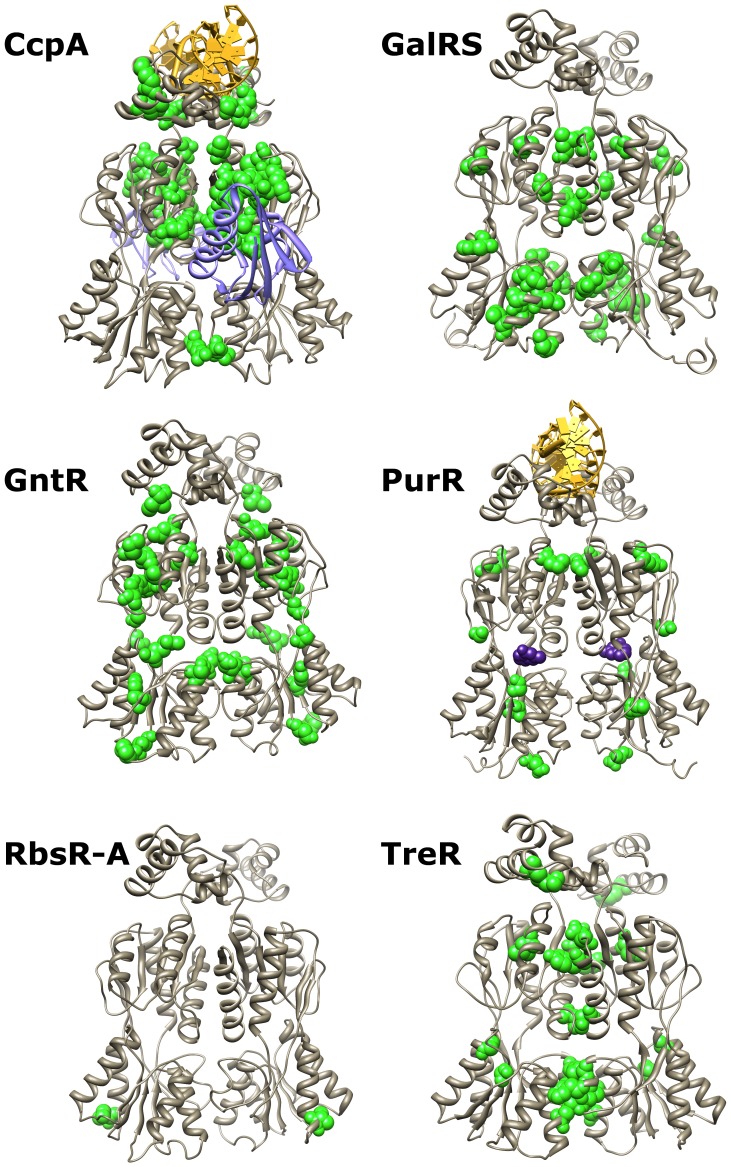
Structural context of subfamily-specific conserved sites. Subfamily-specific conserved positions are shown in green and space-filling on their respective crystallographic or modeled structures. The DNA operator (yellow) is shown bound to CcpA and PurR; an allosteric effector is shown bound to PurR (purple spacefilling); phosphorylated HPr (purple ribbon) is shown bound to CcpA. Allosteric effector binding in the cleft between N- and C-regulatory subdomains is common to all six subfamilies, while HPr-Ser46-P interacts only with CcpA. Molecular graphics were created in UCSF Chimera 1.6.2 [33].

Subfamily-specific conservation is expected if paralogous repressors independently evolved additional protein-protein interactions. Indeed, for the CcpA and GalRS subfamilies, known protein binding sites were highlighted. For the CcpA subfamily, many of the subfamily-specific sites clustered near or in the interface between CcpA regulatory N-subdomain and HPr-Ser46-P [62] (Figure 4, top left; a similar strategy was used to initially identify the CcpA-HPr binding interface [62]). For the GalRS subfamily, the unique tetramerization interface in the regulatory C-subdomain was highlighted by a cluster of subfamily-specific conserved positions [63], [64] (Figure 4, top right). In contrast, the other four subfamilies lack known hetero-protein partners or homo-tetramerization, and subfamily-specific conserved positions were scattered at various locations on the tertiary structure. (Figure 4) This result can be interpreted in at least three ways: (1) other protein-protein interactions do not exist, (2) protein-protein contacts do not require surface conservation, or (3) multiple subfamilies use the same positions for protein-protein interactions. In the absence of additional protein-protein interactions, we hypothesize that the disparate locations of the conserved positions reflects the different ways the LacI/GalR tertiary structure maintains the common functions of DNA binding, ligand binding, allosteric response and/or homodimerization.

### Co-evolutionary Networks differ between LacI/GalR Subfamilies

Co-evolution analyses quantify the evolutionary constraint between every pair of non-conserved amino acids. (By definition, conserved positions cannot co-evolve). High scores indicate strongly correlated mutations, whereas low scores indicate little (or no) co-evolution. To analyze the data, the scores can be organized into an all-vs-all (adjacency) matrix of non-conserved MSA positions. The matrix data are often represented with heat maps, but data can also be treated as networks and analyzed with graph theory. Co-evolution networks (“graphs”) are constructed by connecting protein positions (“nodes” or “vertices”) with edges that are weighted by the strength of their co-evolution scores. The resultant networks can be compared between subfamilies by either (a) calculating the number of preserved edges between the two networks or (b) comparing the set of nodes that are associated with high-scoring edges.

In either format, “important” co-evolving pairs are often designated by imposing some threshold on the data (e.g. the top N% of scores). However, threshold choice is arbitrary; the data seldom (if ever) contain an obvious break between “important” and “non-important” scores. To avoid this problem, we explored how varying the threshold impacted the number of common edges or nodes between any two pairs of subfamilies in the LacI/GalR family. To that end, we iteratively compared the coevolution networks as the threshold varied across the entire range of co-evolution scores (from the top 1% of scores to 100% of scores). For each threshold value, we quantified the similarity of the set of significant edges or nodes by calculating the Jaccard index [65] (“J”). These plots were also compared against models for “perfect agreement” and “random chance”.

**Figure 5:**
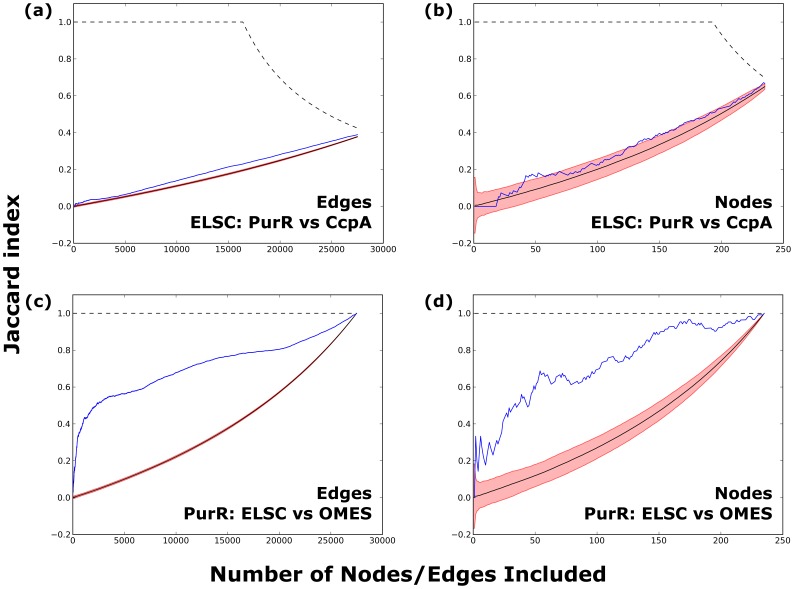
Jaccard analyses. The Jaccard index comparing the set of *N* most highly-scoring edges (panel a) or nodes (panel b) between PurR and CcpA is shown as a function of threshold *N* (blue lines). The example shown is for ELSC; all other algorithms and comparisons to all other subfamilies are in Figures S2–S6 in Data S1 and Figures S18–S22 in Data S4. For reference, plots show the Jaccard indices expected for (i) random overlap (solid black line) ±95% confidence intervals (red region) and (ii) perfect agreement (dotted black line); at large *N*, the line for “perfect agreement” falls below 1 because the PurR and CcpA MSAs do not have identical lengths and the number of conserved positions differ. Comparsion of the PurR and CcpA subfamilies reveals little similarity, though more than expected by chance alone. For comparison, significant similarity was seen for the nodes (c) and edges (d) when results from two algorithms (ELSC and OMES) were compared for one subfamily (PurR). Similar plots and conclusions were obtained when McBASC, OMES, SCA and ZNMI were used to compare all pairs of subfamilies.

We first compared the similarity of high scoring edges for each pair of LacI/GalR subfamilies (Figure 5a and Figures S2–S6 in Data S1). For any given co-evolution algorithm, Jaccard similarity indices for the LacI/GalR subfamilies were much more similar to the random model (solid black line; 95% confidence intervals shown in red) than to the agreement model (dotted black line). Importantly, this observation held for *all* threshold values. In comparison, analyses of one subfamily with alternative algorithms yielded considerably higher agreement (Figure 5c). Therefore, although the similarity of edges is statistically distinguishable from what would be expected from random chance alone, the co-evolution network of each subfamily was highly dissimilar from those of other subfamilies. One exception was found in the comparison of the PurR and RbsR-A subfamilies, which showed slightly more agreement than other subfamily pairs (Figures S2–S6 in Data S1). This result likely reflects their higher sequence similarity (45% between the *E. coli* paralogs) as compared to other subfamily pairs (∼17–31%) (Figure 2a). Nevertheless, the RbsR-A and PurR results still fit much more closely to the random model than to the agreement model.

**Figure 6:**
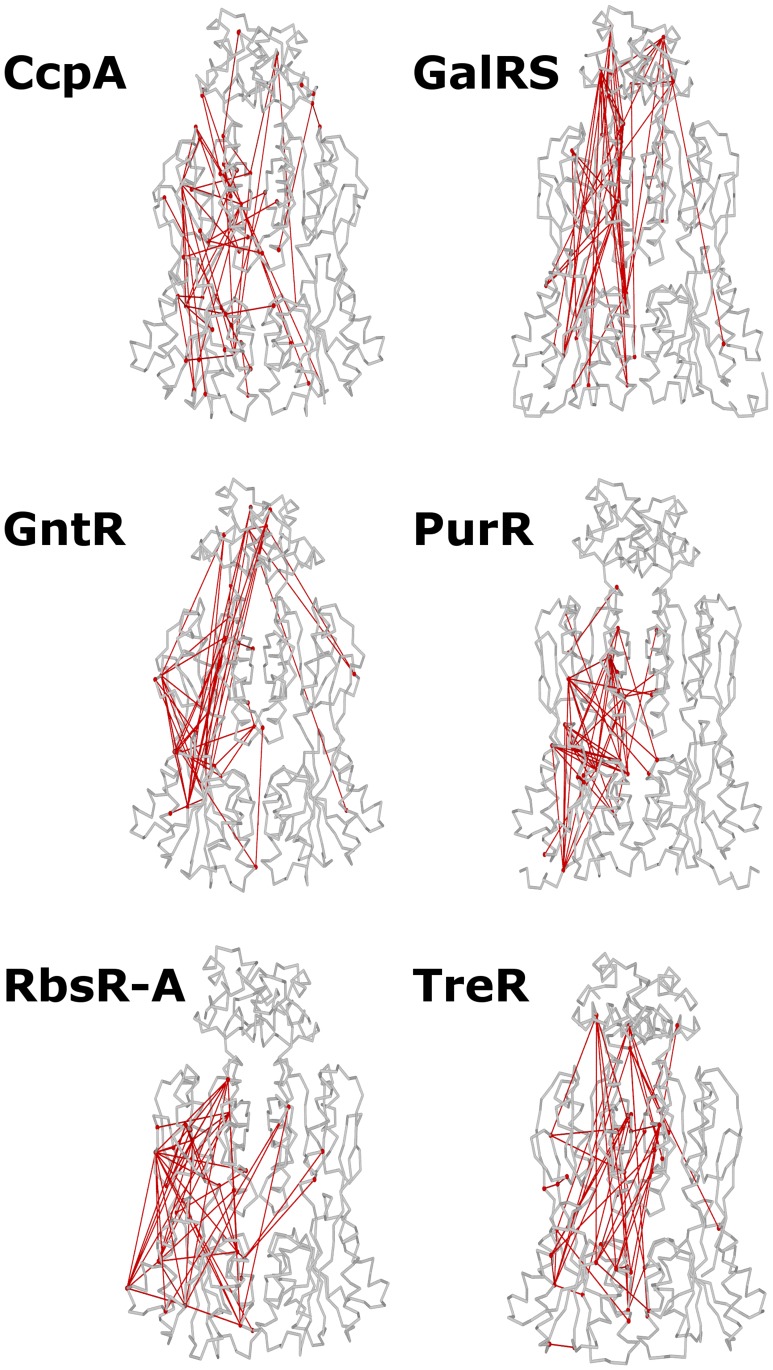
Top co-evolving edges for each subfamily. The 50 highest scoring edges for each of the six subfamilies’ ELSC networks (CcpA, a; GalRS, b; GntR, c; PurR, d; RbsR-A, e; TreR; f) were mapped onto the backbone trace of the full-length crystal (Ccpa, PurR) or ITASSER model structures (GalRS, GntR, RbsR-A, TreR). Edges are drawn only once on each structure: between the position in the left monomer and its closest partner on either the left or right monomer (not both). The pattern of spatial connectivity differed greatly among subfamilies. Molecular graphics were created using PovRay 3.7 (Persistence of Vision Pty. Ltd., Williamstown, Victoria, Australia; http://www.povray.org) and custom software.

**Figure 7:**
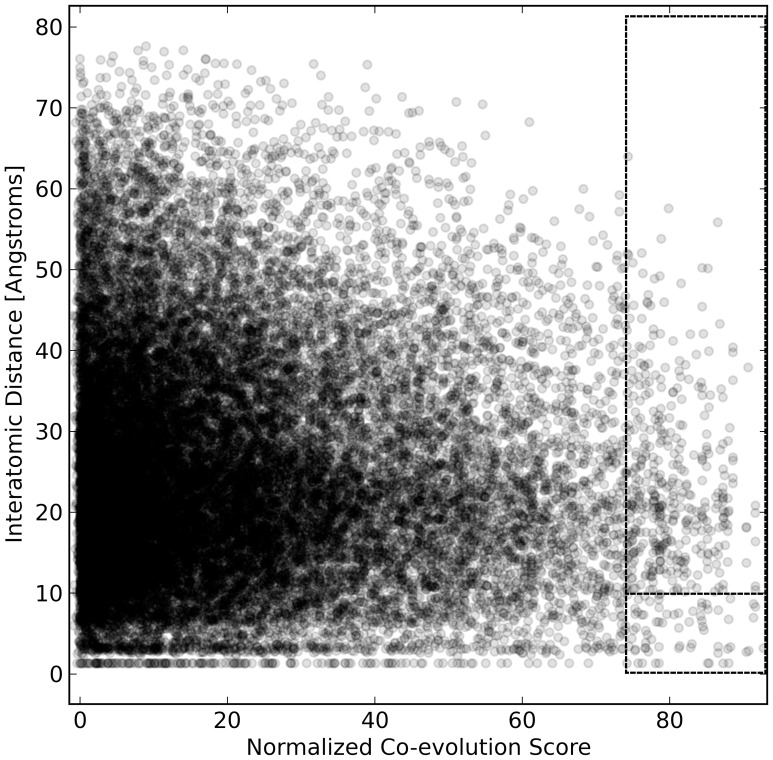
3D structural contacts do not correlate with co-evolution scores. The poor correlation between inter-atomic distance and (Z-normalized) co-evolution score is shown for one example subfamily/algorithm pair (PurR/ELSC). Points are plotted with 15% opacity to aid visual inspection. Highly co-evolving pairs of positions are often spatially distant (upper dotted-line box), rather than proximal (lower dotted-line box). Other subfamily/algorithm pairs show a similar absence of correlation (Figures S12–S17 in Data S3).

To illustrate the divergent co-evolution networks obtained for each subfamily, we mapped the 50 highest scoring edges onto representative tertiary structures (Figure 6 and Figures S7–S11 in Data S2). Most strongly co-evolving pairs of positions were distant in tertiary structure. Indeed, analysis of all edges revealed virtually no correlation between co-evolution score and proximity in 3D space (Figure 7 and Figures S12–S17 in Data S3). These results are consistent with recent experimental reports of epistasis (non-additivity) between positions that are spatially distant on the LacI structure [66].

Similar analyses were also performed to compare strongly co-evolving nodes. The amino acid positions (nodes) were rank-ordered by their strongest edge values and Jaccard analyses were performed for all possible threshold values for all pairwise comparisons of the LacI/GalR subfamilies. As with edge-based analyses, nodal comparisons were much closer to the random model than to the agreement model (Figure 5b and Figures S18–S22 in Data S4). Indeed, comparing the nodal Jaccard indices to the 95% confidence region of the random model reveals that the similarity of most subfamily pairs is essentially *indistinguishable* from the random model for virtually all threshold values. As was the case in edgewise analysis, the RbsR-A and PurR subfamilies showed slightly more similarity than random chance, but the comparison was still far from the agreement model.

**Figure 8:**
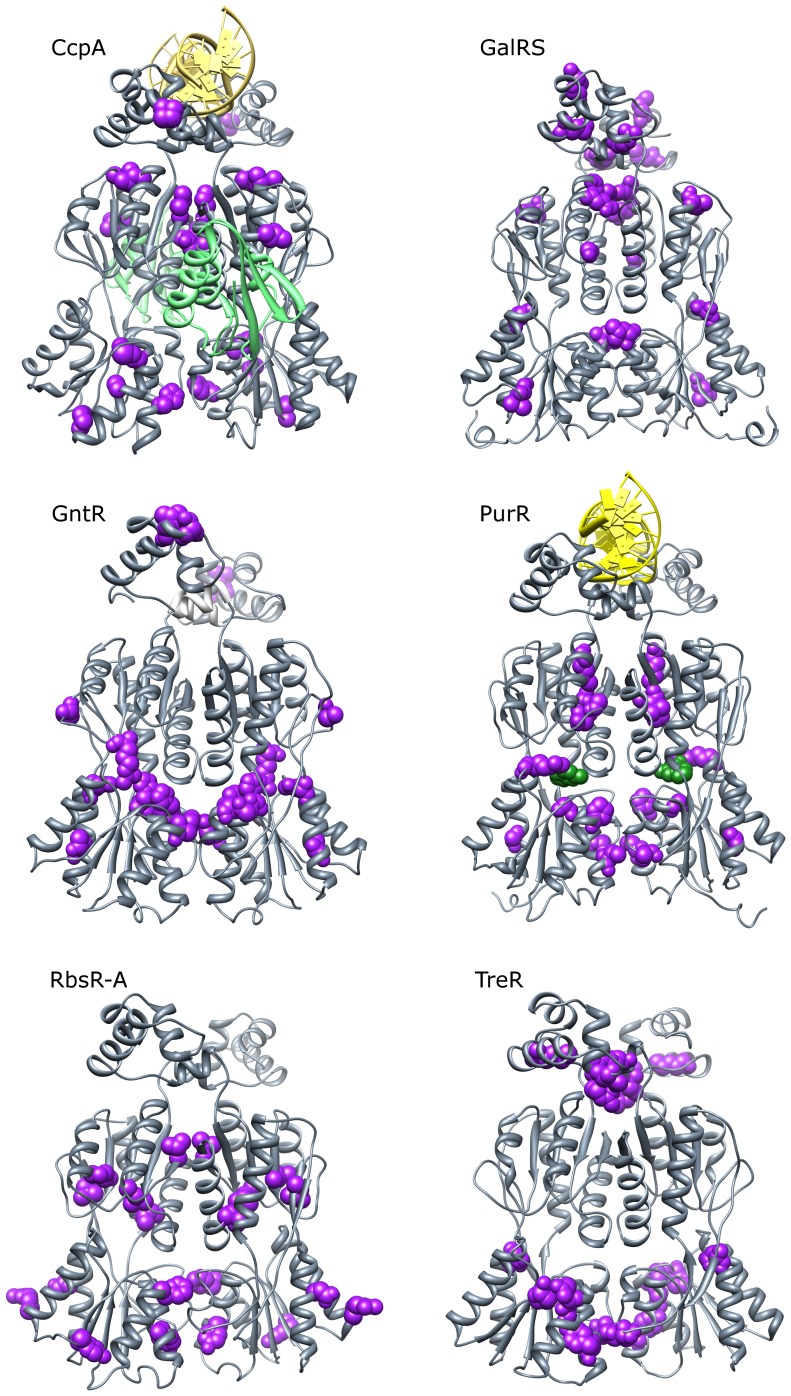
Top co-evolving positions for each subfamily. Positions (nodes) were scored by the weight of their largest edge in each of the 5 co-evolution networks (ELSC, OMES, McBASC, ZNMI, SCA). These scores were Z-normalized and averaged across the algorithms. Positions were ranked by their average Z-score and the top 10 positions (purple spacefilling) were plotted onto full-length crystal structures (CcpA, PurR) or ITASSER models (GalRS, GntR, RbsR-A, TreR). The DNA operator (yellow) is shown bound to CcpA and PurR; an allosteric effector is shown bound to PurR (green spacefilling); phosphorylated HPr (green ribbon) is shown bound to CcpA. Allosteric effector binding in the cleft between N- and C-regulatory subdomains is common to all six subfamilies, while HPr-Ser46-P interacts only with CcpA. Note that the locations of highly co-evolving sites were not consistent among subfamilies. Molecular graphics were created in UCSF Chimera 1.6.2 [33].

To illustrate the subfamily-specific positions on representative structures, we determined a “consensus” set of highly co-evolving positions using data from all of the co-evolution algorithms (Table 2 and Figure 8; see Methods). As expected from Jaccard analyses, most of the top co-evolving positions were unique to each subfamily; no top nodes were identified in more than three subfamilies (Table 2, bold). Among the 50 highly co-evolving positions across the six subfamilies, only two were identified in three subfamilies (to enable comparisons, the LacI numbering system is used): position 163 in GalRS, GntR and TreR and position 222 in GntR, PurR and RbsR-A. Position 222 is located at the inter-monomer dimerization interface, but to our knowledge, the functional and structural roles of position 163 have not been tested in the relevant subfamilies. However, position 163 was implicated by targeted molecular dynamics as a key residue involved in the allosteric transition of LacI [67].

**Table 2:** Structural features associated with top co-evolving positions.

		Top 10 co-evolving node in…							
LacI #	Domain*	CcpA	GalRS	GntR	PurR	RbsR-A	TreR	Structural Contact	
7	D		X					DNA	
25	D						X	DNA	
31	D			X				DNA	
32	D	X							
34	D			X				DNA	
**42**	D		**X**				**X**		
51	L						X	Dimer interface	
**54**	L		**X**				**X**	**DNA**	
66	R				X				
68	R				X				
79	R					X		Ligand	
84	R	X						Dimer interface	
**95**	R	**X**				**X**		Dimer interface	
97	R				X			Dimer interface	
99	R		X						
113	R		X					Dimer interface	
114	R				X				
115	R	X						Dimer interface	
117	R		X					Dimer interface	
125	R					X		Ligand	
127	R			X					
143	R	X							
147	R					X			
154	R			X					
158	R					X			
**163**	R		**X**	**X**			**X**		
168	R					X			
**171**	R			**X**	**X**				
191	R				X			Ligand	
207	R					X			
**214**	R		**X**			**X**			
218	R						X		
**222**	R			**X**	**X**	**X**		**Dimer interface**	
224	R						X		
225	R						X		
228	R	X							
239	R	X							
246	R				X			Ligand	
249	R	X							
254	R					X			
**255**	R	**X**			**X**			**Dimer interface**	
256	R						X		
274	R			X				Ligand	
275	R			X					
276	R			X					
277	R		X					Dimer interface	
283	R						X	Dimer interface	
285	R				X			Dimer interface	
303	R	X						HPr Binding Site	
307	R		X						

Domains: D, DNA binding domain; L, Linker; R, Regulatory domain.

Surprisingly few of the other 48 top co-evolving positions have been subjected to mutagenesis in the relevant subfamily. To assess their biological significance, we therefore turned to structural analyses, reasoning that positions involved in protein-protein, protein-DNA, or protein-ligand interactions should be critical for LacI/GalR function. Of the 50 top co-evolving positions identified across the six subfamilies, 23 (46%) were located in structurally-important locations: Twelve were located at the dimeric interface, five were in contact with DNA, five were in contact with the allosteric effector and one of the CcpA positions was located at the HPr-Ser46-P binding site. Thus, the common tertiary structure appears to utilize different locations to facilitate the core repressor functions of homodimerization, ligand binding, and allosteric signaling.

Taken together, these results strongly suggest that the co-evolutionary networks are biologically important but highly dissimilar between subfamilies. This result is not dependent on the details of any one co-evolution algorithm: all five methods revealed the same features.

### GalR and GalS Isorepressors have Divergent Co-evolution Networks

In the prior descriptions of the LacI/GalR sequences [14], we noted deeper levels of sequence clustering within each subfamily (Figure 2b). Given the subfamily-specific evolutionary patterns, we were curious whether subclusters that occur within one subfamily showed similar or divergent evolutionary patterns. The GalRS subfamily has five subclusters, and the *E. coli* GalR and GalS iso-repressors fall into two of them. GalR and GalS are encoded by separate genes at distinct loci, yet both regulate the *gal* operon by binding to the same DNA operator and respond to the same allosteric effector [17]. *E. coli* GalR and GalS show 54% sequence identity to each other, which is significantly larger than between most subfamilies (generally <40%).

**Figure 9:**
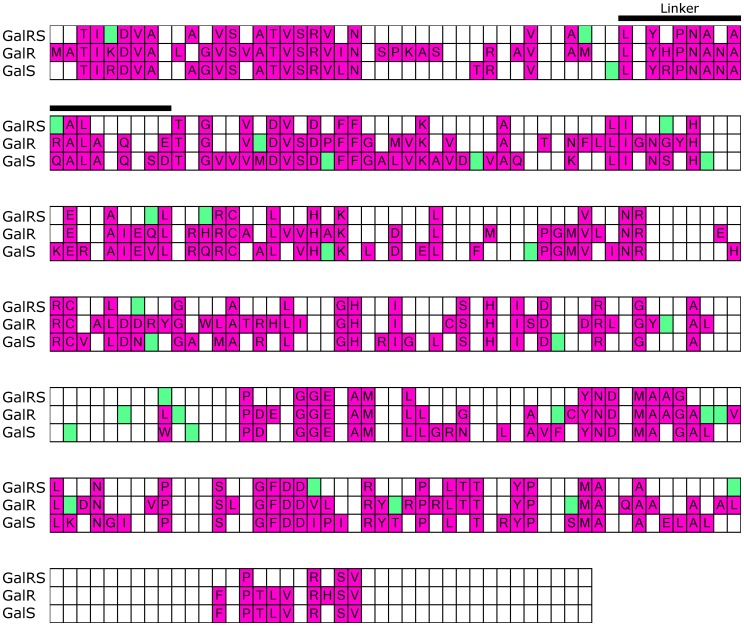
Conserved and co-evolving positions in GalRS. Conserved positions (magenta cells) in the entire GalRS subfamily (top line), the GalR subset (middle line), or GalS subset (bottom line) are shown on the alignment. The amino acid shown in conserved cells is the most common amino acid in the respective subfamily. The top 10 co-evolving positions for the GalRS subfamily and each subset are shown in green.

We first determined that several conserved positions in the GalR and GalS subclusters were found at the positions that were conserved or co-evolving in the larger GalRS subfamily. About 40% of positions (148 of 346) are conserved in both the GalR and GalS subclusters. An additional 90 positions (26%) were conserved in only one of the two subclusters. Of the ten most strongly co-evolving positions for the GalRS subfamily, eight were conserved in both subsets, whereas two (position 42 and 307) were conserved only in GalR. Thus, strongly co-evolving positions in the entire GalRS (Figure 9, green cells on top line) subfamily are those at which different types of amino acids are required in each of the isorepressors.

Nevertheless, co-evolution analyses for the separate GalR and GalS subclusters again showed divergent networks. Jaccard index analyses of both key edges and nodes in the co-evolution networks were dissimilar for the GalR and GalS subclusters (Figures S23–S25 in Data S5). Therefore, even when further constrained to perform the same DNA binding function under the same allosteric regulation, iso-repressors are not required to have similar evolutionary constraints at non-conserved positions. As a caveat for this particular example, we note that GalR tetramerization and DNA looping might have a role in chromosomal organization [68] that has not been documented for GalS; this could be the source for some of the altered evolutionary pressure.

## Discussion

To investigate alternative mechanisms of protein evolution, we have compared the subfamily-specific conservation patterns and co-evolutionary networks among several LacI/GalR subfamilies. One evolutionary possibility is that the location of key non-conserved positions is “hard-wired” into their common tertiary structure. If so, then a single set of conserved and highly co-evolving positions should be preserved amongst different subfamilies and adaptation would be accomplished by varying the amino acid at those sites. On the other hand, the tertiary scaffold might accommodate multiple conservation patterns and co-evolution networks. In this case, adaptation could be accomplished by varying both the choice of amino acid and the loci at which variation occurs. Our results showed that evolution utilized both the “hard-wired structure” and the “accommodating scaffold” framework for subfamily-specific conservation in the LacI/GalR proteins (Figures 3 and 4). In contrast, the accommodating scaffold framework was overwhelmingly utilized for subfamily-specific co-evolution networks (Figures 5, 6, 8 and 9).

A question of both theoretical and practical interest is: By what mechanism do alternative co-evolution networks arise? Phylogenetic analyses of the LacI/GalR family showed that the DNA binding domain fused with the regulatory domain only once, before the “divergence of the major lineages of eubacteria” [40] (2500–4000 million years [69]). Thus, new repressor variations must have resulted from functional divergence from a single repressor. In such cases, a common assumption is that the subsequent proteins evolved from a state of ancestral low-specificity to modern high-specificity [70], [71]. If this is the case, then the ancestral LacI/GalR repressor could have served as a blank template. Varied selection pressures would then result in the development of alternative co-evolution networks.

However, some protein families do not appear to fit this model; for example, the ancestral steroid receptor had ligand specificity similar to that of modern-day estrogen receptors [72]. Additionally, a small number of large-effect mutations were sufficient to significantly shift the ancestral protein’s specificity profile [73]. Thus, an alternative possibility for the LacI/GalR subfamilies is that they rapidly diverged (i.e., using only a few mutations) from a high-specificity ancestral repressor. Unfortunately, the ancestral LacI/GalR protein cannot be resurrected because this family has been diverging for billions of years and the phylogenetic tree lacks intermediate sequence information.

Nevertheless, there is no *a priori* reason to believe that the network of co-evolving sites in the LacI/GalR progenitors must be reflected in present-day subfamiles. Indeed, if ancestral reconstruction could be accomplished, it might generate sequences that recapitulate specificity but lack optimization. The latter might require optimization of the subfamily-specific co-evolution network. For example, although the resurrected ancestral steroid receptor specifically binds to estrogen, affinity is 2500x-fold lower than its modern counterpart [72]. This reduction in specificity could be either (a) a true feature of the ancestral receptor, or (b) an artifact of reconstruction that did not account for an accommodating tertiary scaffold featuring alternative, functionally important sites. To incorporate the “accommodating scaffold” model into analyses, highly co-evolving sites in the immediate downstream lineages of an ancestral protein should be considered as potentially important, especially when validating the statistical support [74] for the choice of amino acid in the reconstructed sequence.

Our results also have implications for interpreting other sequence analyses of modern proteins. In addition to co-evolution, dozens of algorithms have been developed to predict functionally-important sites from protein MSAs (e.g. [9], [15], [75]–[79] among others). Predictions are (i) highly dependent on the sequences used to construct the MSA and (ii) limited to patterns that persist throughout the entire dataset. Our data suggests that some sites may be functionally-important in only a small number of subfamilies (or, indeed, a single subfamily or subcluster) (Figures 3 and 4). These positions would not be detected by algorithms that look for patterns that persist across the entire family (e.g. Figure 3, asterisk and daggers).

To make best use of existing tools, both the whole family and individual subfamilies (if a sufficient number of sequences are available) should be analyzed separately. This type of “multi-level” MSA analysis identified additional functionally-important sites in the psychoactive bioamine G protein-coupled receptors [80] and should be widely employed. Finally, analyses of mixed ortholog-paralog MSAs should *not* assume that the functional roles of non-conserved positions are consistent across subfamilies, which is especially important when mutational results are extrapolated from one homolog to another.

A third outcome of our study is the observation that ? for all 6 subfamilies ? the strongest co-evolution occurs between spatially distinct positions (Figures 6 and 7). Prior analyses of other proteins have suggested that co-evolution predicts spatial proximity [41]–[46]. However, some of the prior studies were performed with proteins much smaller than the LacI/GalR proteins, which by itself increases the chance of identifying close neighbors. Instead, our results are consistent with (i) other studies that have shown that the accuracy for predicting spatial contacts is <30% [46] and (ii) long-range experimental epistasis recently reported for LacI, which shares a common tertiary scaffold with the proteins of this study [66]. The sparse spatial distributions of highly co-evolving positions might arise if these positions influence protein dynamics, altering the distribution of conformations sampled by the transcription regulators.In summary, multiple co-evolutionary networks are supported by the common tertiary scaffold of the LacI/GalR proteins, and individual subfamilies show different conservation patterns. These observations support the idea of an adaptive scaffold that can evolve on top of the “hard-wired” structural and functional framework that is present in for the whole family. Future computational analyses should be improved by accounting for the functionally-important, non-conserved positions that are unique to each subfamily.

## Methods

### Subfamily Selection and Expansion

We previously reported that a set of 1344 LacI/GalR homologs clusters into at least 34 subfamilies [14]. Several new structures reported as part of the Protein Structure Initiative [22]–[32] nucleated an additional 11 subfamilies (unpublished data). Each subfamily has high internal sequence identity (>40%) but there is low sequence identity between subfamilies (typically <30%) (Figure 2) [14] Of these, we chose the six largest subfamilies for co-evolution analysis: CcpA, GalRS, GntR, PurR, RbsR-A, and TreR (Table 1; note that all are part of the published data set). Each subfamily was expanded to include sequences that were added to the RefSeq database since the initial compilation of the LacI/GalR dataset in May 2009 [14] to the cutoff date for this study, May 2011.

New sequences were retrieved using representative sequences for each subfamily as a seed sequences in a BLAST [81] search of RefSeq [82]. To verify newly retrieved sequences, we used MUSCLE [83] to add the new sequences to existing MSAs of LacI/GalR family members and verified that the sequence most closely matched the relevant subfamily [14]. Once the final sequences were identified, MUSCLE was used to construct final alignments for each subfamily. Although the MSA for the complete LacI/GalR family required manual editing benchmarked against structure-based alignments [14], the subfamily MSAs could be appropriately constructed with MUSCLE because of the high internal sequence identities. Final subfamily alignments were manually inspected to verify their reasonability. These alignments are available upon request. Maximum likelihood phylogenetic trees were calculated with RAxML 7.0.3 using the default parameters and the PROTGAMMABLOSUM62 substitution model [84].

One criterion for reliable MSA analyses is that very closely related sequences (such as one branch of a phylogenetic tree) are not over-represented. To show the phylogenetic distribution of each subfamily, we constructed maximum-likelihood phylogenetic trees (Figure S1 in Data S1). The resulting trees have a stellate appearance, indicating that divergent lineages are sampled and that no single protein sequence or bacterial species was over-represented. A second criterion for reliable MSA analyses is that the sequence set is large enough to avoid statistical errors that arise from low sampling rates. We analyzed the reproducibility of our results to verify that: (a) a sufficient number of sequences were present in each subfamily to prevent sampling bias and (b) any alignment error in individual sequences did not influence the co-evolution analyses; details of these analyses are listed below.

### Designation and Alignment of Reference Sequences

Reference sequences were designated for the GalRS, GntR, PurR, RbsR-A, and TreR subfamilies by identifying the homolog from *E. coli str. K-12 substr. MG1655*, as annotated by RefSeq Release 47 (May 07, 2011) [82]. *E. coli* does not contain an ortholog of CcpA, so the homolog from *Bacillus subtilis subsp. subtilis str. 168* was designated as the reference sequence. In order to map amino acid positions to the LacI reference numbering system, the sequence for *E. coli str. K-12 substr. MG1655* LacI was included in the reference set.

Reference sequences were initially aligned using Promals3D,which yields higher quality alignments of distantly related sequences than other tools [85]. The alignment of reference sequences was benchmarked against available crystal structures (PDB: 1efa:A, 1wet:A, 1byk:A, 1rzr:A and our previous alignment [14]); the reference alignment was manually optimized in light of available data [18]–[21]. This alignment is available in Table S2 in Data S5. The average length of the reference sequences was 332 residues. The longest reference sequence contained 343 residues (CcpA), while the shortest contained 315 residues (TreR). The reference alignment contained 354 columns.

### Co-evolution Analysis

Co-evolution analyses were performed using the implementation of ELSC [43], OMES [47], [54] McBASC [44], [55], [56] and SCA [51] algorithms available from Fodor *et al.* [43], [54] To reconcile differences in output format and to improve speed, we re-implemented ZNMI in C# and validated this version against the Python-based implementation described by Brown and Brown [53]. For validation, the RbsR-A subfamily was used as a test dataset. This re-implementation of ZNMI, and other software (Figures S26–S27 and Table S1 in Data S5), is available at https://sourceforge.net/projects/coevolutils/.

Details of the ELSC, OMES, and ZNMI algorithms were excellently reviewed by Brown and Brown [53]. As previously described [14], we modified the Fodor *et al.*’s source code to remove filtration of 90% identical sequences from McBASC. For each subfamily, ensembles comprising 100 different subsets of the alignment with 90% or 50% of the available sequences were constructed by random sub-sampling. The co-evolution algorithms were applied to each sub-alignment and the score for each pair of position was averaged across each ensemble to produce a final score. Positions that included more than 50% gaps, following [54], or less than 5% sequence variability (which was imposed as a minimum value on column-wise sequence entropy [53], namely – [.05 ln(.05) +.95 ln(.95)] ≈ 0.1985), were excluded from further analyses. Positional entropy was calculated for each sequence using the equation.
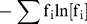
(1)where f_i_ is the frequency for each of the i'th type of amino acid (Ala, Ile, Leu, etc.), including gaps as a “21^st^ amino acid”. The exception was the ZNMI algorithm, which internally calculates positional entropy ignores all gaps, a convention we retained for compatibility with the original implementation of the algorithm. Additionally, Brown and Brown’s ZNMI algorithm excludes all positions with >10% gaps, a convention we also retained for compatibility with their implementation. Pairwise co-evolution scores were mapped onto the reference sequence for each subfamily to facilitate comparison between subfamilies using the reference alignment and to allow mapping to the LacI numbering system, which is commonly used for the LacI/GalR family.

To validate the MSAs for co-evolution analyses, we used a modified version of the subsample-and-reanalyze cross-validation strategy [50], [53]. For each LacI/GalR subfamily, two ensembles of 100 subalignments were created. One ensemble comprised subalignments that randomly selected 90% of the sequences; the second ensemble comprised subalignments that randomly selected 50% of the sequences. Analyses were carried out for each subalignment in the ensemble, and the 100 scores were averaged to generate a final score. This strategy reduced the influence of alignment errors that might arise from any one (or small group of) sequence(s). In virtually all cases, the average co-evolution score of the 90% ensemble exhibited extremely strong correlations with the average score for the 50% ensemble (median Pearson correlation coefficient (R) = 0.996), which demonstrated that: (a) any alignment error in individual sequences did not substantially influence the generated scores and (b) a sufficient number of sequences were present in each subfamily to prevent sampling bias. Consequently, all analyses were carried out using only the results from analysis of the 90% ensemble. The lowest correlations were observed in the GalR- and GalS-only subsets when analyzed with McBASC. However, GalR- and GalS-only McBASC scores still exhibited strong correlations between the 90% and 50% ensembles (Pearson R = 0.928 and 0.876, respectively). The GalR and GalS alignments contain the fewest number of sequences of any alignment used in this study, and thus are most vulnerable to finite sampling effects.

To calculate the “consensus set” of high scoring nodes for each subfamily, we first assigned each position a score equal to the maximum weight of its edges. A “consensus” score was then assigned to each position by averaging the maximum-edge scores produced by each of the algorithms. Before averaging, the maximum-edge scores were Z-normalized (*i.e.* made to have equal mean and variance) to ensure that no one algorithm dominated the consensus score by virtue of assigning a larger range of raw scores.

### Molecular Modeling and Structural Analysis

The LacI/GalR proteins are homodimers, with each monomer comprising a DNA binding domain linked to a large regulatory domain [18], [20], [21]. The DNA binding domains have higher sequence identity across the whole family, whereas the regulatory domains show more divergence [14], [16]. Of the subfamilies in this study, full-length crystal structures were available for the CcpA (PDB: 1rzr) [20] and PurR (PDB: 1wet) [21] subfamilies. A regulatory-domain structure was available for the TreR subfamily (PDB: 1byk) [19]. We created full-length structural models for the GntR, RbsR-A, TreR, and GalRS subfamilies using I-TASSER [86] with default parameters; note that neither DNA nor allosteric ligands were present in the modeling process. As a test of the I-TASSER models, the crystal structure of the TreR regulatory domain [19] was aligned with the model. The comparison had an RMSD of 2.055 Å for the alpha carbons, with a few deviations observed in loops between secondary structure elements. Thus, the homology model is adequate for visualization purposes.

For structural analyses, contacts between co-evolving positions with DNA and across the dimeric interface were defined if they were present in any of the all-atom representations of the full-length crystal structures (PDB: 1efa, LacI; 1rzr, CcpA; 1wet, PurR) or the TreR regulatory domain-only structure (PDB: 1byk) [18]–[21]. Contacts at the effector binding site were defined if they were present in any crystallographic structures with effectors bound (PDB: 1efa, LacI; 1byk, TreR; 1wet, PurR; 2nzv, CcpA) [18], [19], [21], [87]. Atomic contacts were defined on the basis of inter-atomic distance, using the same criteria as the default parameters of Resmap [88]. DNA-protein and protein-protein contacts were obtained from Resmap analyses. Contacts between the proteins and their allosteric effector ligands were obtained by custom software implementing the Resmap contact criteria, in order to specify new hetero-atomic contacts. In this case, this Resmap configuration file was modified to include the atomic classes (hydrophilic, acceptor, donor, hydrophobic, aromatic, neutral, neutral-donor, neutral-acceptor) of the ligand heteroatoms after parsing their assignments from results from the Ligand-Protein Contacts and Contacts of Structural Units (LPC/CSU) analyses [89].

### Network Construction and Comparison

Co-evolution networks were constructed as an undirected, weighted graph with nodes denoting positions and edges weighted according to the co-evolution score assigned to pairs of positions. Data were further analyzed using SciDAVis (http://scidavis.sourceforge.net/), Prism 5.04 (GraphPad Software Inc., La Jolla, CA), Network Workbench (http://nwb.cns.iu.edu/), and custom software implemented in C#, Python and Bash (Figures S26–S27 and Table S1 in Data S5). Several scientific python libraries were used, including NetworkX, SciPy and NumPy. The Jaccard index (*J*) for similarity of sets [65], A and B, containing *N* elements of which *k* elements occur as members of both sets was calculated using the formula:
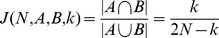
(2)The null-model probability distribution of obtaining *k* shared elements by randomly drawing *N* elements from each of two sets, A and B, with |A∩B| = θ can be exactly calculated, but is computationally expensive when comparing sets containing a large number of elements. Therefore, we approximated the mean and standard deviation of the null-model distribution by sampling *J(N,A,B,k)* across 1000 shufflings of the rank-ordered lists of high scoring nodes/edges. The 95% confidence interval of the random model was calculated by multiplying the standard deviation of this sampling by 1.96.

## Supporting Information

Data S1 **Phylogenetic trees and edgewise Jaccard analyses.** Figure S1. Phylogenetic trees of the six subfamilies. Maximum likelihood trees for each of the six subfamilies were calculated with RAxML 7.0.3 using the default parameters and the PROTGAMMABLOSUM62 substitution model. Trees universally have a stellate appearance indicating the subfamilies include sequences from a variety of microbial lineages. Figures S2–S6. Edgewise Jaccard analyses for all pairs of subfamiles. The Jaccard index for the set of *N* most highly-scoring pairs of positions (edges), using each algorithm (figures), between all pairs of the six subfamilies (panels) is shown as a function of *N* (a-e, blue lines). The expected Jaccard index under the random model (black line, solid), 95% confidence interval of the expected index (red region), and maximum possible Jaccard index (black line, dotted) are shown.
(PDF)

Data S2 **Co-evolving edges mapped to structure.** Figures S7–S11. Structurally-mapped co-evolution networks. The 50 highest scoring edges for each of the six subfamilies' networks is shown mapped onto the backbone trace of the full-length crystal (Ccpa, PurR) or ITASSER model structures (GalRS, GntR, RbsR-A, TreR). Alternative figures represent alternative co-evolution algorithms. High-scoring co-evolving edges are drawn only once on the structure: between the residue in the left monomer and its partner in either the left or right monomer (not both), so as to minimizes the inter-atomic distance spanned by the edge. The pattern of spatial connectivity is not consistent across subfamilies (see Results, Jaccard analysis). Molecular graphics were created using PovRay 3.7 (Persistence of Vision Pty. Ltd., Williamstown, Victoria, Australia; http://www.povray.org) and custom software.
(PDF)

Data S3 **3D structural proximity vs score.** Figures S12–S17. Lack of 3D structural proximity. The lack of correlation between inter-atomic distance and co-evolution score is shown for each algorithm (panels), applied to each subfamily (figures). Points are plotted with 15% opacity to aid in visualization. Strength of co-evolution score does not correlate with inter-atomic distance. The marginal distributions (counts) of inter-atomic distances (right) and co-evolution scores (top) are displayed as histograms.
(PDF)

Data S4 **Nodal Jaccard analyses.** Figures S18–S22. Nodal Jaccard analyses for all pairs of subfamilies. The Jaccard index for the set of *N* most highly-scoring positions (nodes), using each algorithm (figures), between all pairs of the six subfamilies (panels) is shown as a function of *N* (a-e, blue lines). The expected Jaccard index under the random model (black line, solid), 95% confidence interval of the expected index (red region), and maximum possible Jaccard index (black line, dotted) are shown.
(PDF)

Data S5 **GalR versus GalS and software pipeline.** Figure S23. The GalR and GalS isorepressors: edgewise Jaccard analysis. The similarity of the set of *N* most highly co-evolving edges in the GalR and GalS isorepressors, as a function of the threshold (*N*) is shown (blue line). Comparison to the expected and 95% confidence interval of the random overlap model (black line and red region) and the perfect agreement model (black dotted line) are shown. Subpanels delineate comparisons made using different co-evolution analysis algorithms (ELSC, OMES, McBASC, SCA and ZNMI). Figure S24. The GalR and GalS isorepressors: nodal Jaccard analysis. The similarity of the set of *N* most highly co-evolving nodes in the GalR and GalS isorepressors, as a function of the threshold (*N*) is shown (blue line). Comparison to the expected and 95% confidence interval of the random overlap model (black line and red region) and the perfect agreement model (black dotted line) are shown. Subpanels delineate comparisons made using different co-evolution analysis algorithms (ELSC, OMES, McBASC, SCA and ZNMI). Figure S25. The GalR and GalS isorepressors: Highly co-evolving positions mapped to the structure. The 10 most strongly co-evolving positions in the GalR (spacefilled green) and GalS (spacefilled magenta) isorepressors are shown on the ITASSER model structure for GalR. Molecular graphics were created with UCSF Chimera. Figure S26. Analytical workflow, overview. Figure S27. Analytical workflow, ensemble-based co-evolution analysis. Table S1. Description of available programs. Table S2. Reference sequence alignment.
(PDF)
