# Supplementary material for: Multiple Co-Evolutionary Networks Are Supported by the Common Tertiary Scaffold of the LacI/GalR Proteins
Source: PLoS One. 2013 Dec 31;8(12):e84398. doi: 10.1371/journal.pone.0084398 (PMC3877293; doi:10.1371/journal.pone.0084398)
Supplement: Data S1 — Phylogenetic trees and edgewise Jaccard analyses. Figure S1. Phylogenetic trees of the six subfamilies. Maximum likelihood trees for each of the six subfamilies were calculated with RAxML 7.0.3 using the default parameters and the PROTGAMMABLOSUM62 substitution model. Trees universally have a stellate appearance indicating the subfamilies include sequences from a variety of microbial lineages. Figures S2–S6. Edgewise Jaccard analyses for all pairs of subfamiles. The Jaccard index for the set of N most highly-scoring pairs of positions (edges), using each algorithm (figures), between all pairs of the six subfamilies (panels) is shown as a function of N (a-e, blue lines). The expected Jaccard index under the random model (black line, solid), 95% confidence interval of the expected index (red region), and maximum possible Jaccard index (black line, dotted) are shown. (PDF) [file pone.0084398.s001.pdf]

Supplemental data for:
Multiple co-evolutionary networks are supported by the common tertiary scaffold of the LacI/GalR proteins
File 1: Phylogenetic trees and edgewise Jaccard analyses

Daniel J. Parente and Liskin Swint-Kruse

List of Figures

S1 Phylogenetic trees of the six subfamilies . . . 2
S2 Edgewise Jaccard analyses, all pairs of subfamilies: ELSC . . . 4
S3 Edgewise Jaccard analyses, all pairs of subfamilies: OMES . . . 5
S4 Edgewise Jaccard analyses, all pairs of subfamilies: McBASC . . . 6
S5 Edgewise Jaccard analyses, all pairs of subfamilies: SCA . . . 7
S6 Edgewise Jaccard analyses, all pairs of subfamilies: ZNMI . . . 8

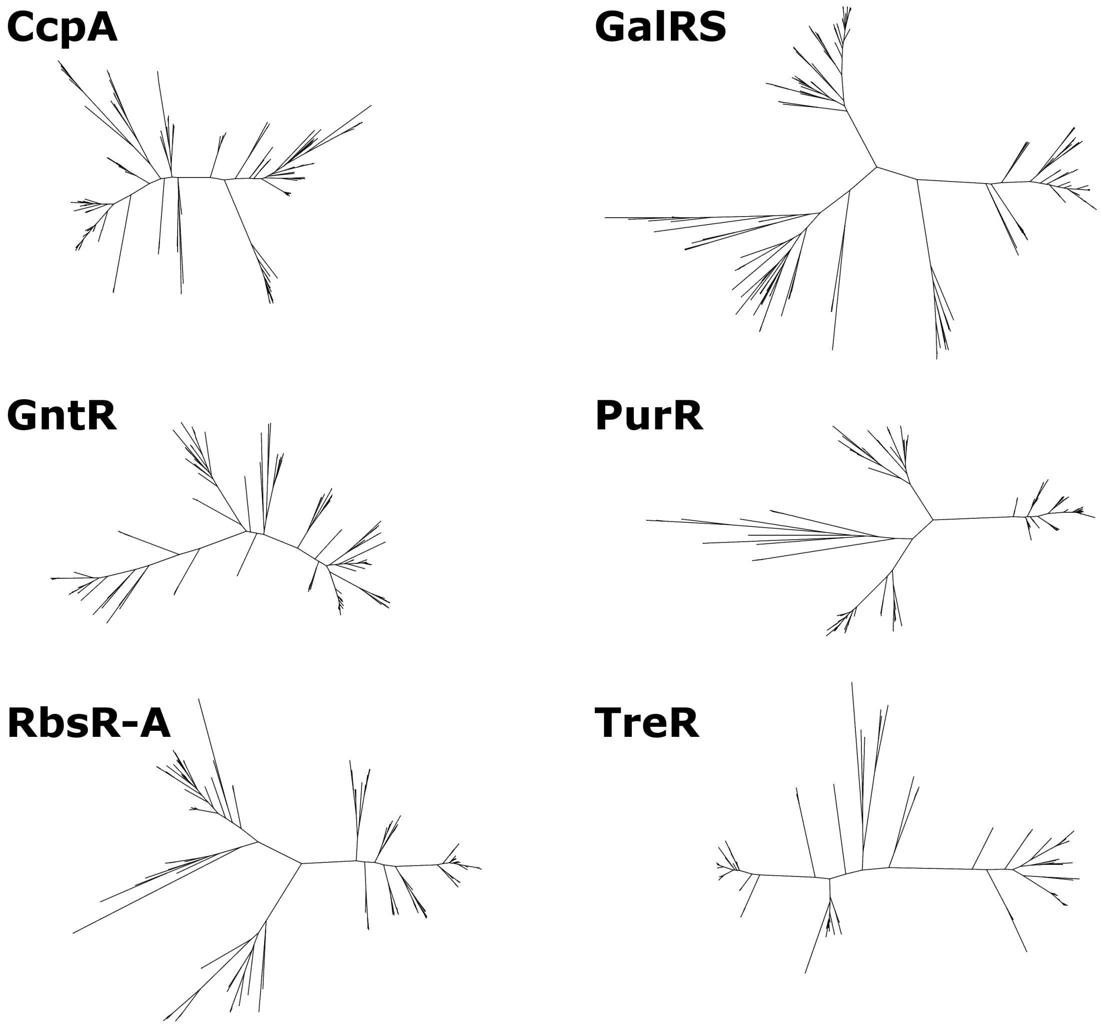

Figure S1: Phylogenetic trees of the six subfamilies. Maximum likelihood trees for each of the six subfamilies were calculated with RAxML 7.0.3 using the default parameters and the PROTGAMMABLOSUM62 substitution model. Trees universally have a stellate appearance indicating the subfamilies include sequences from a variety of microbial lineages.

Figures S2-S6: Edgewise Jaccard analyses for all pairs of subfamiles. The Jaccard index for the set of N most highly-scoring pairs of positions (edges), using each algorithm (figures), between all pairs of the six subfamilies (panels) is shown as a function of N (a-e, blue lines). The expected Jaccard index under the random model (black line, solid), 95% confidence interval of the expected index (red region), and maximum possible Jaccard index (black line, dotted) are shown.

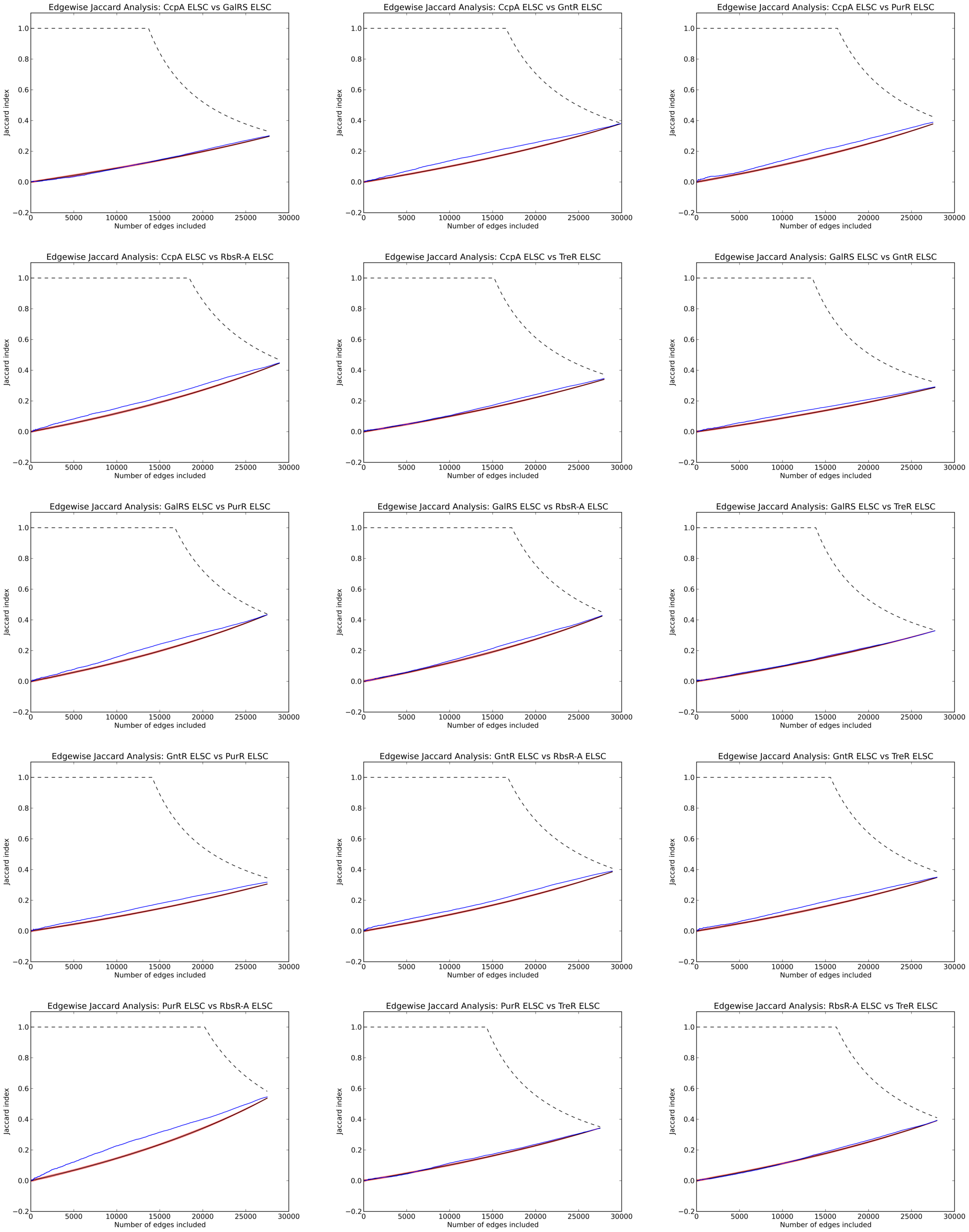

Figure S2: Edgewise Jaccard analyses, all pairs of subfamilies: ELSC

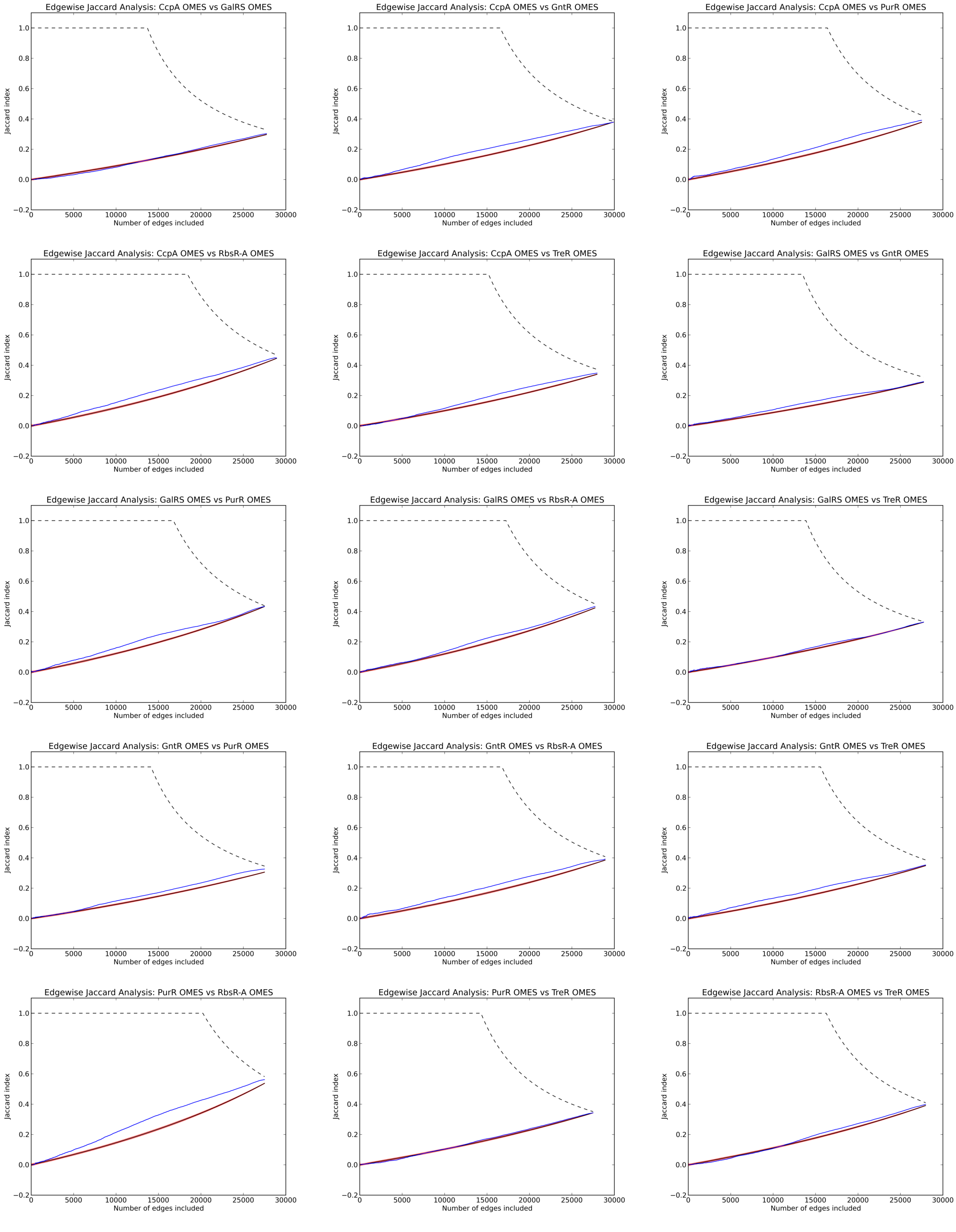

Figure S3: Edgewise Jaccard analyses, all pairs of subfamilies: OMES

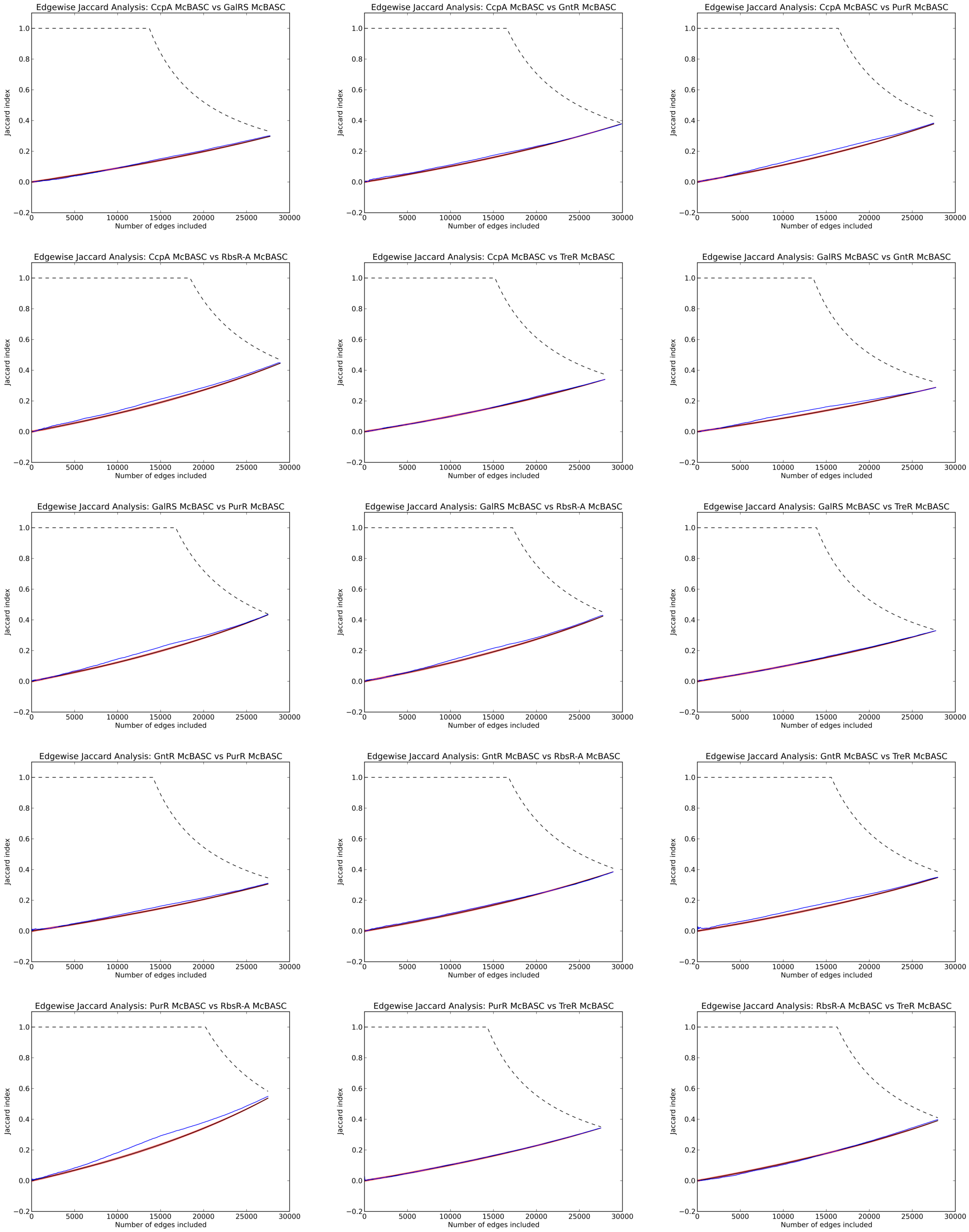

Figure S4: Edgewise Jaccard analyses, all pairs of subfamilies: McBASC

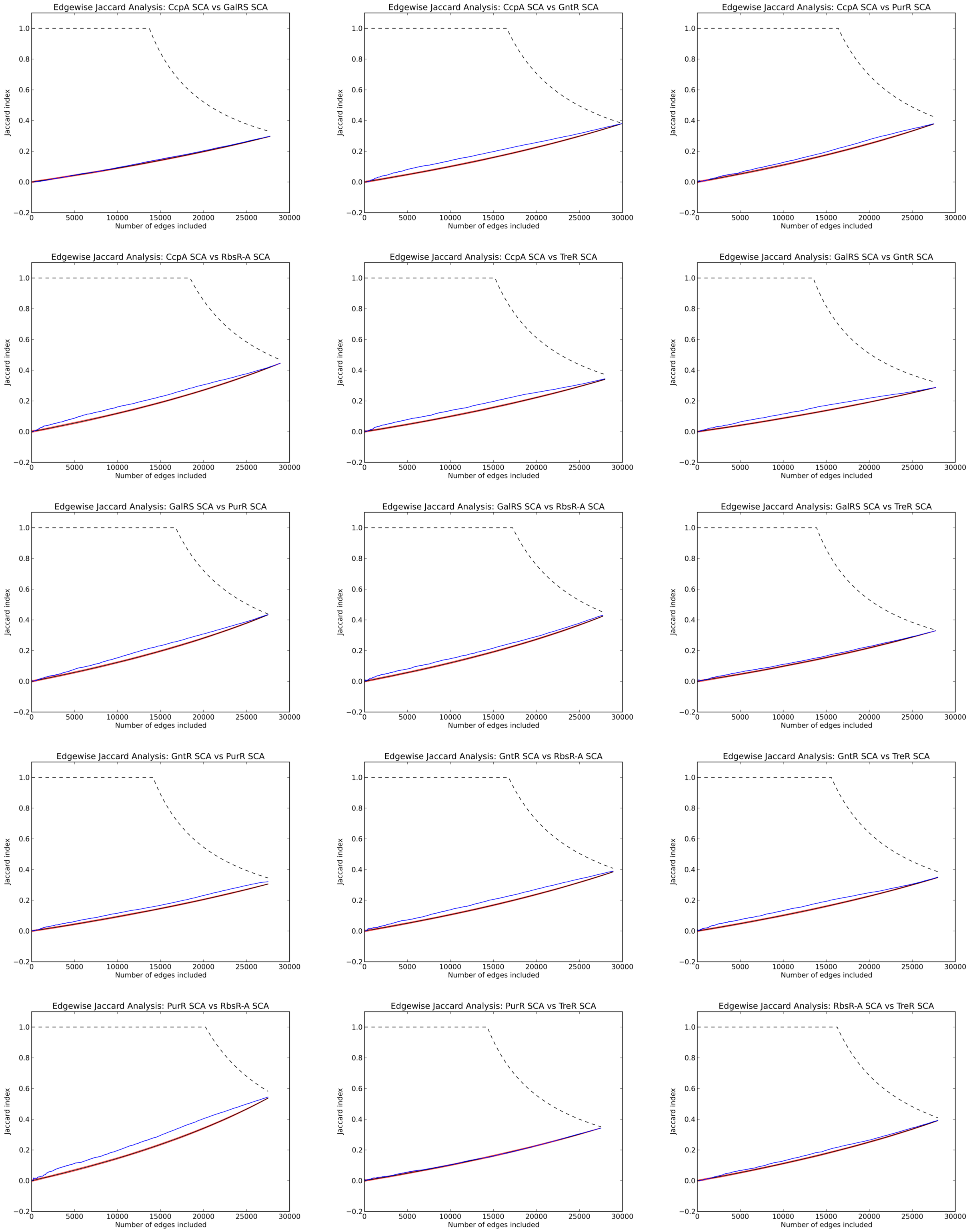

Figure S5: Edgewise Jaccard analyses, all pairs of subfamilies: SCA

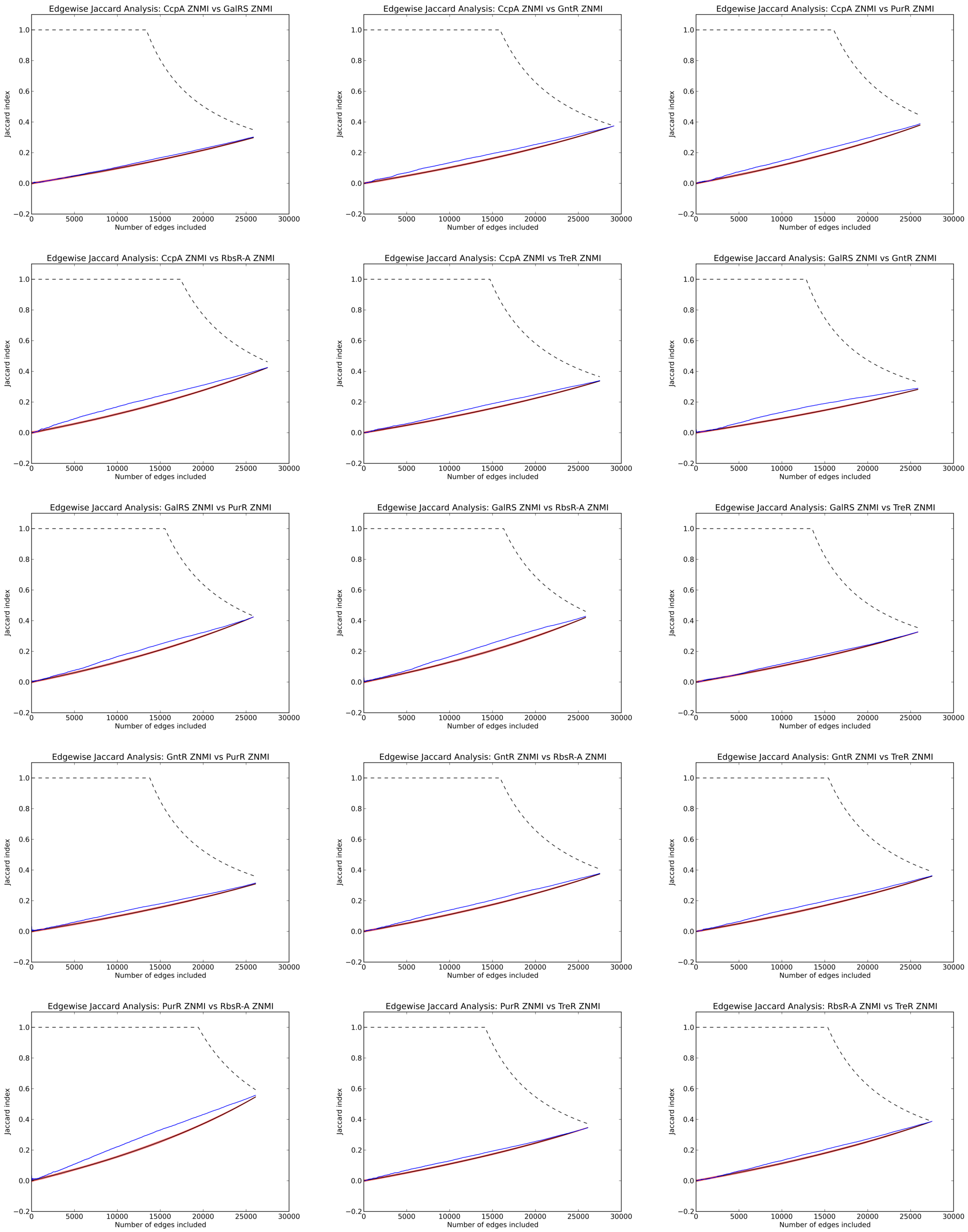

Figure S6: Edgewise Jaccard analyses, all pairs of subfamilies: ZNMI
