# Supplementary material for: Multiple Co-Evolutionary Networks Are Supported by the Common Tertiary Scaffold of the LacI/GalR Proteins
Source: PLoS One. 2013 Dec 31;8(12):e84398. doi: 10.1371/journal.pone.0084398 (PMC3877293; doi:10.1371/journal.pone.0084398)
Supplement: Data S2 — Co-evolving edges mapped to structure. Figures S7–S11. Structurally-mapped co-evolution networks. The 50 highest scoring edges for each of the six subfamilies' networks is shown mapped onto the backbone trace of the full-length crystal (Ccpa, PurR) or ITASSER model structures (GalRS, GntR, RbsR-A, TreR). Alternative figures represent alternative co-evolution algorithms. High-scoring co-evolving edges are drawn only once on the structure: between the residue in the left monomer and its partner in either the left or right monomer (not both), so as to minimizes the inter-atomic distance spanned by the edge. The pattern of spatial connectivity is not consistent across subfamilies (see Results, Jaccard analysis). Molecular graphics were created using PovRay 3.7 (Persistence of Vision Pty. Ltd., Williamstown, Victoria, Australia; http://www.povray.org) and custom software. (PDF) [file pone.0084398.s002.pdf]

Supplemental data for: Multiple co-evolutionary networks are supported by the common tertiary scaffold of the LacI/GalR proteins File 2: Co-evolving edges mapped to structure

Daniel J. Parente and Liskin Swint-Kruse

List of Figures

S7 Structurally-mapped co-evolution networks: ELSC 3
S8 Structurally-mapped co-evolution networks: OMES 4
S9 Structurally-mapped co-evolution networks: McBASC 5
S10 Structurally-mapped co-evolution networks: SCA 6
S11 Structurally-mapped co-evolution networks: ZNMI 7

Figures S7-S11: Structurally-mapped co-evolution networks. The 50 highest scoring edges for each of the six subfamilies' networks is shown mapped onto the backbone trace of the full-length crystal (Ccpa, PurR) or ITASSER model structures (GalRS, GntR, RbsR-A, TreR). Alternative figures represent alternative co-evolution algorithms. High-scoring co-evolving edges are drawn only once on the structure: between the residue in the left monomer and its partner in either the left or right monomer (not both), so as to minimizes the inter-atomic distance spanned by the edge. The pattern of spatial connectivity is not consistent across subfamilies (see Results, Jaccard analysis). Molecular graphics were created using PovRay 3.7 (Persistence of Vision Pty. Ltd., Williamstown, Victoria, Australia; http://www.povray.org) and custom software.

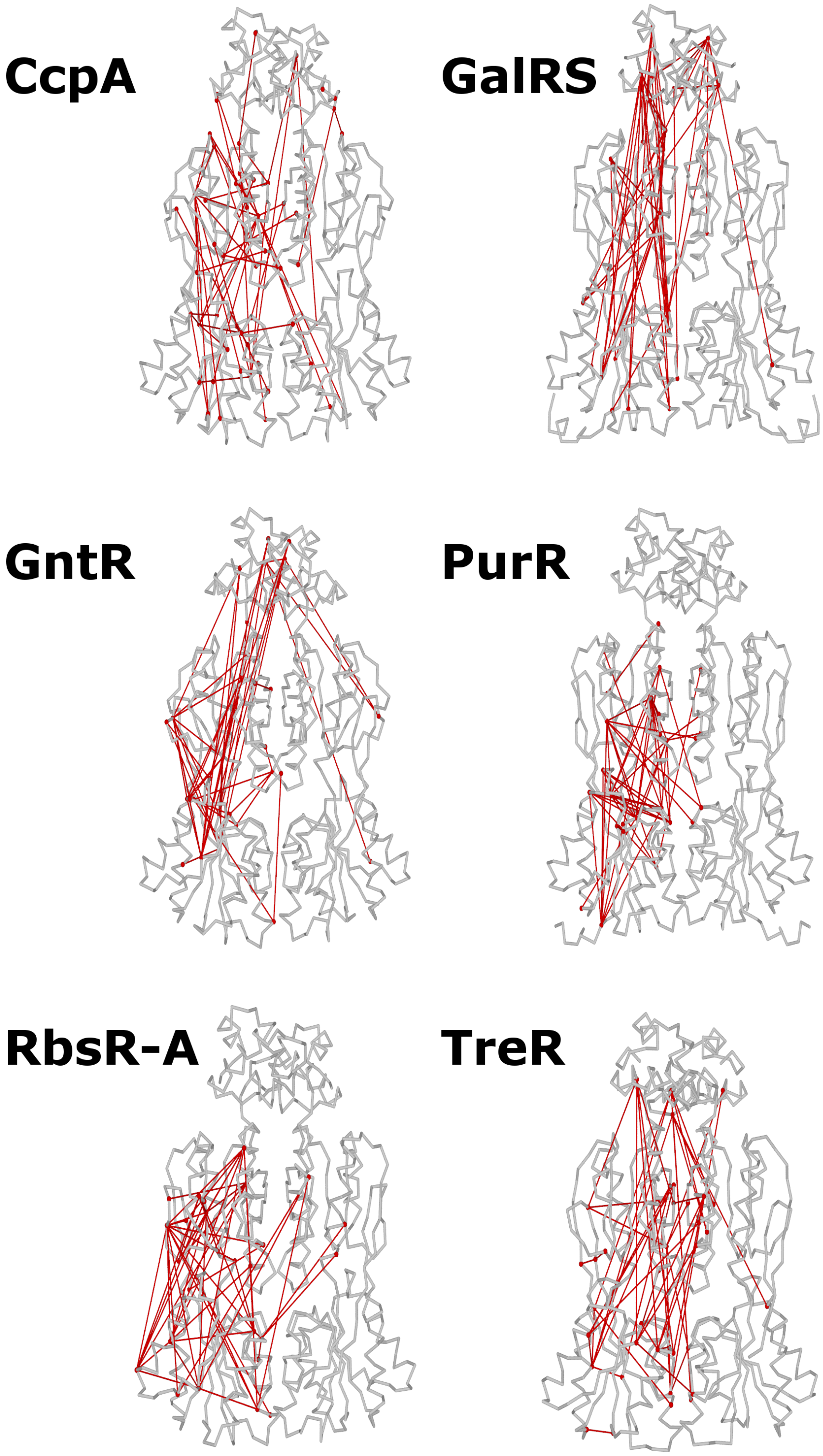

Figure S7: Structurally-mapped co-evolution networks: ELSC

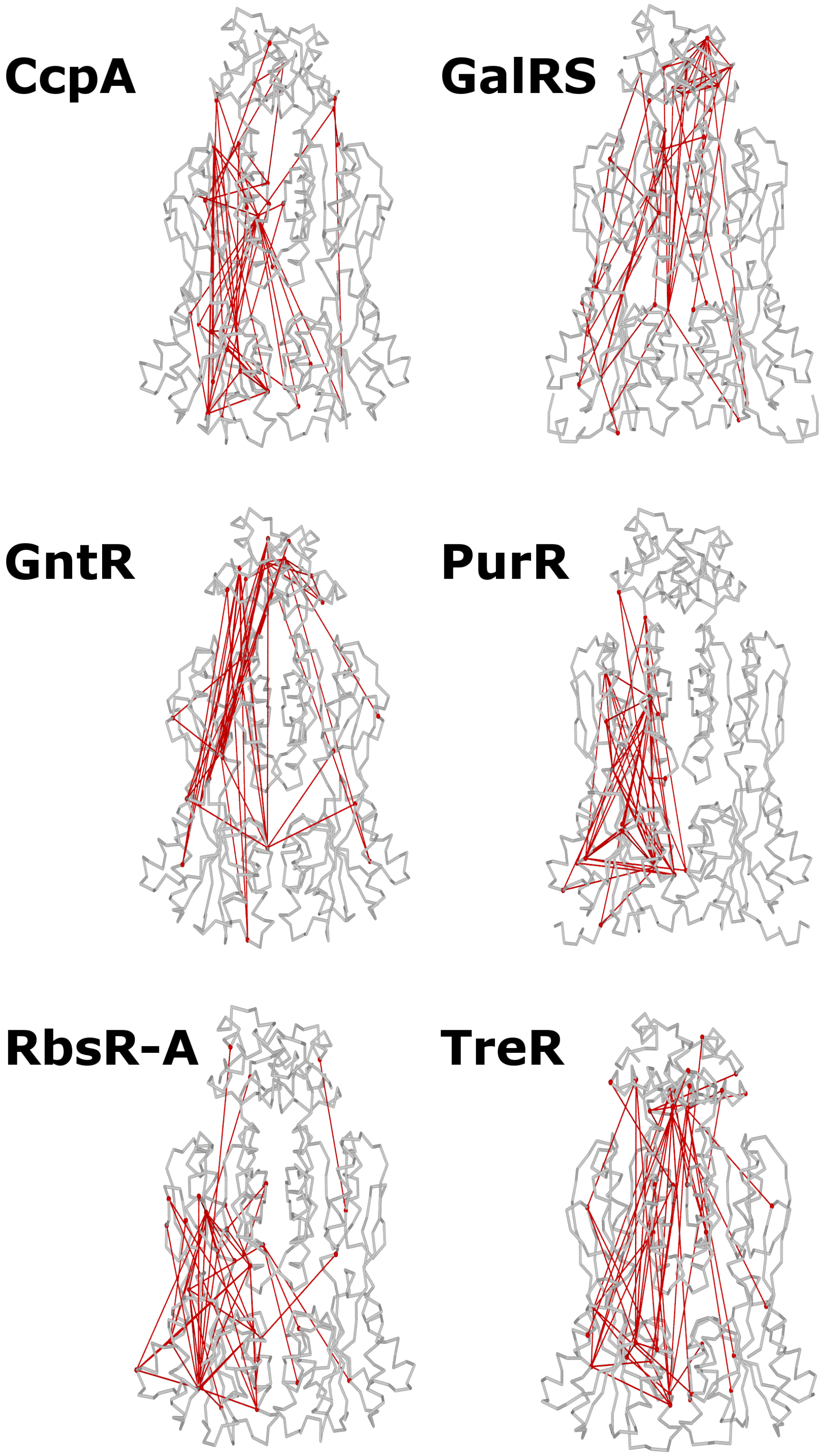

Figure S8: Structurally-mapped co-evolution networks: OMES

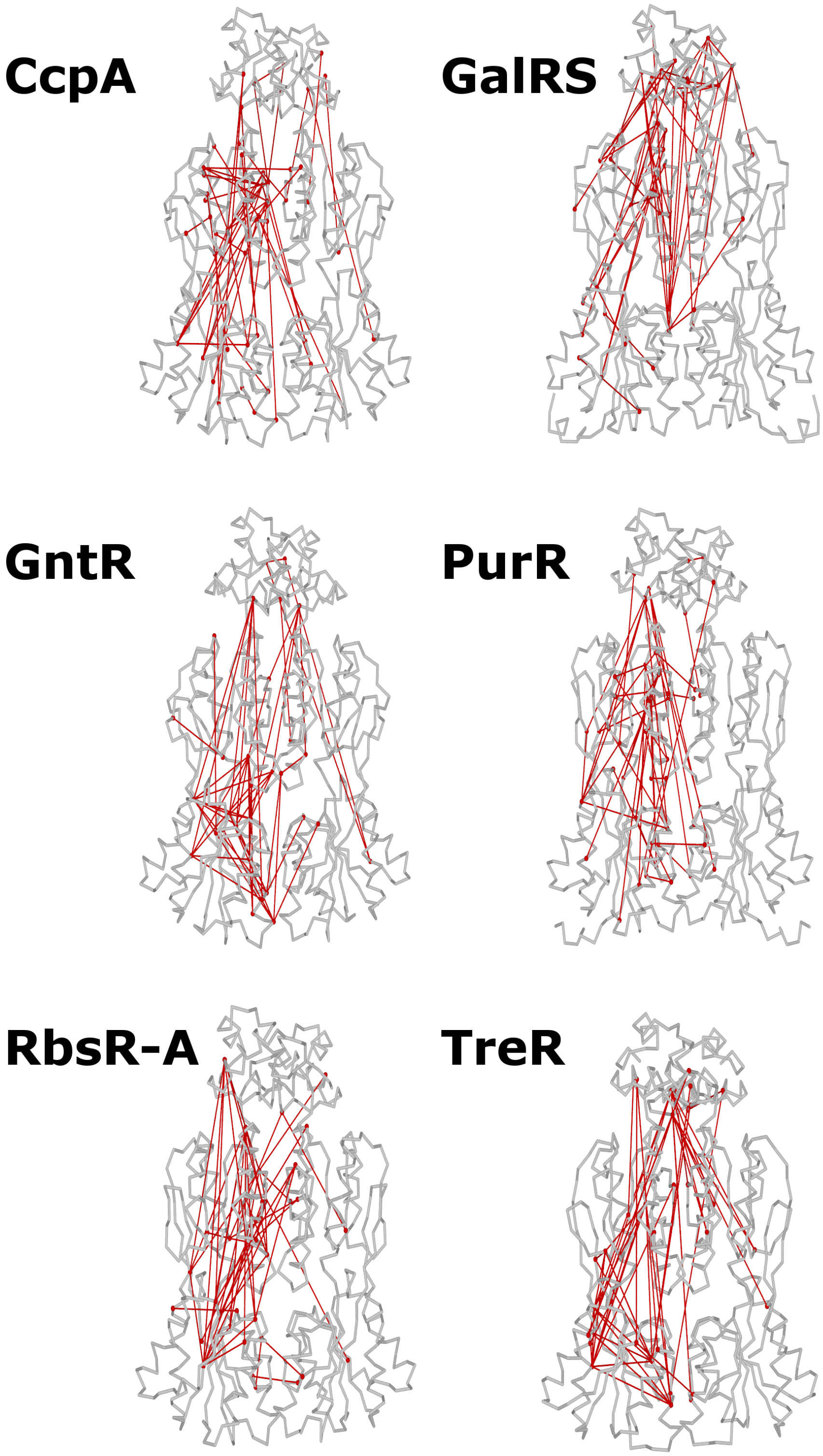

Figure S9: Structurally-mapped co-evolution networks: McBASC

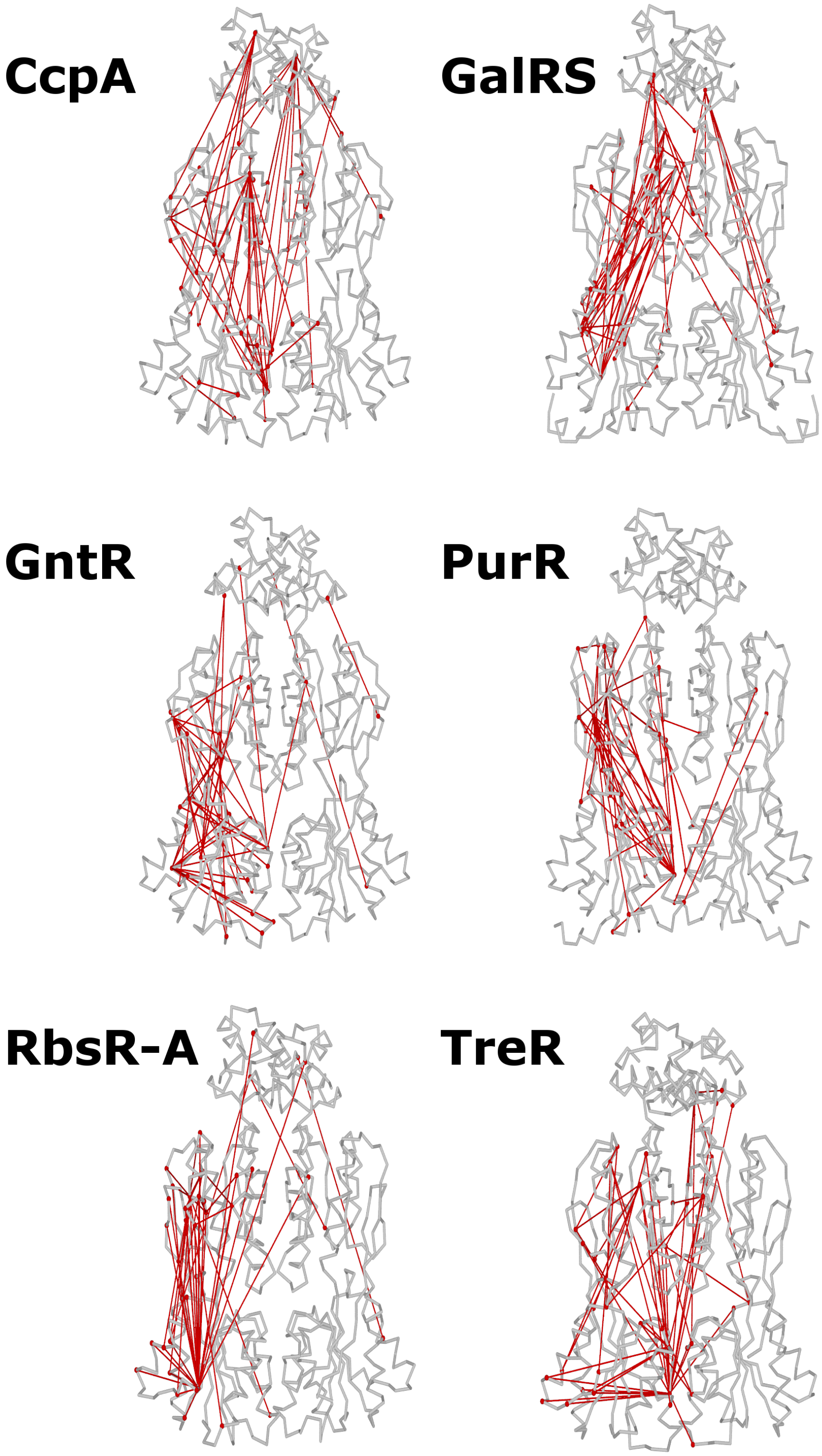

Figure S10: Structurally-mapped co-evolution networks: SCA

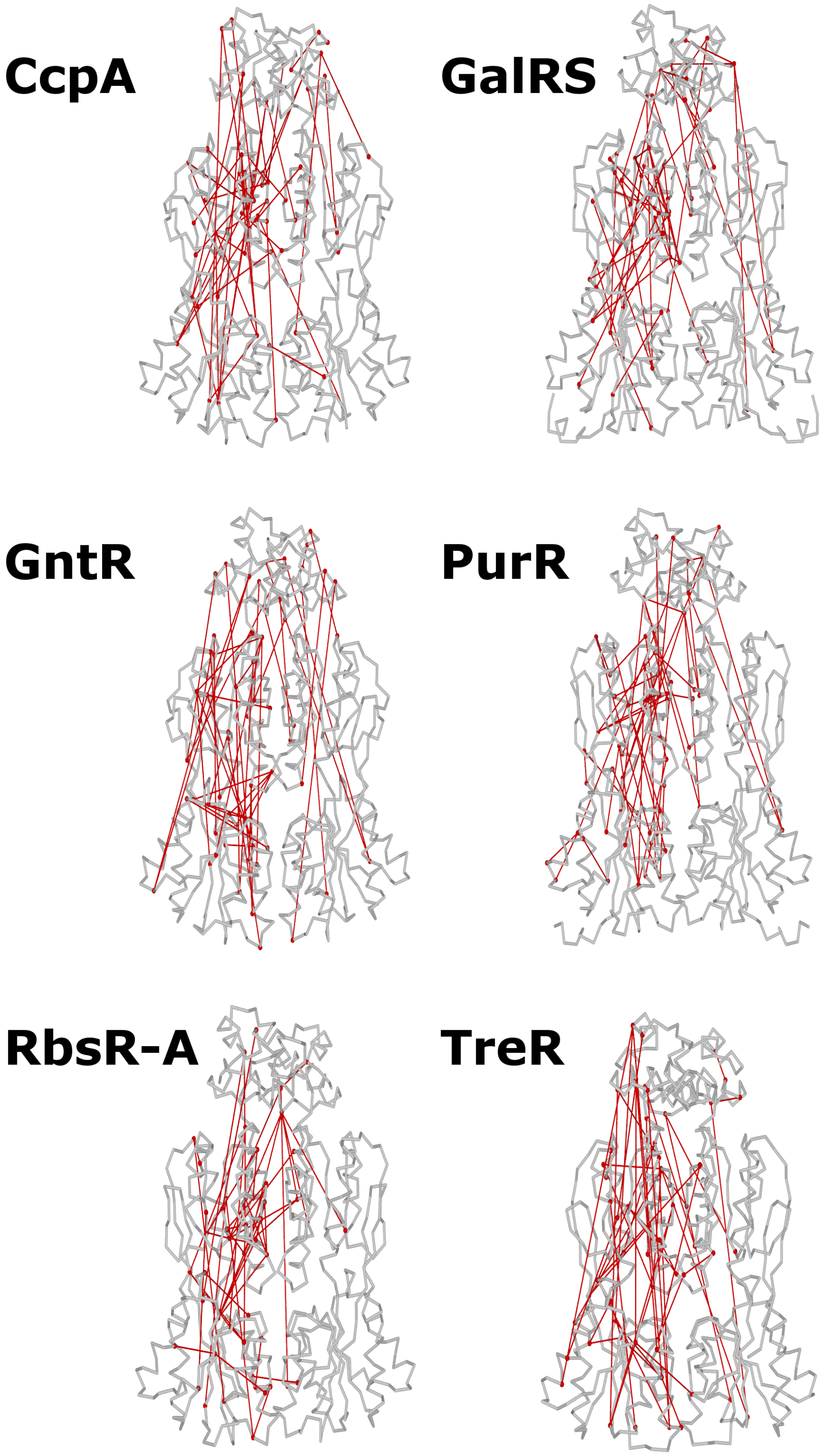

Figure S11: Structurally-mapped co-evolution networks: ZNMI
