# Supplementary material for: Multiple Co-Evolutionary Networks Are Supported by the Common Tertiary Scaffold of the LacI/GalR Proteins
Source: PLoS One. 2013 Dec 31;8(12):e84398. doi: 10.1371/journal.pone.0084398 (PMC3877293; doi:10.1371/journal.pone.0084398)
Supplement: Data S3 — 3D structural proximity vs score. Figures S12–S17. Lack of 3D structural proximity. The lack of correlation between inter-atomic distance and co-evolution score is shown for each algorithm (panels), applied to each subfamily (figures). Points are plotted with 15% opacity to aid in visualization. Strength of co-evolution score does not correlate with inter-atomic distance. The marginal distributions (counts) of inter-atomic distances (right) and co-evolution scores (top) are displayed as histograms. (PDF) [file pone.0084398.s003.pdf]

Supplemental data for:
Multiple co-evolutionary networks are supported by the common tertiary scaffold of the LacI/GalR proteins
File 3: 3D structural proximity vs score

Daniel J. Parente and Liskin Swint-Kruse

List of Figures

S12 Lack of 3D structural proximity: CcpA 3
S13 Lack of 3D structural proximity: GalRS 4
S14 Lack of 3D structural proximity: GntR 5
S15 Lack of 3D structural proximity: PurR 6
S16 Lack of 3D structural proximity: RbsR-A 7
S17 Lack of 3D structural proximity: TreR 8

Figures S12-S17: Lack of 3D structural proximity. The lack of correlation between inter-atomic distance and co-evolution score is shown for each algorithm (panels), applied to each subfamily (figures). Points are plotted with 15% opacity to aid in visualization. Strength of co-evolution score does not correlate with inter-atomic distance. The marginal distributions (counts) of inter-atomic distances (right) and co-evolution scores (top) are displayed as histograms.

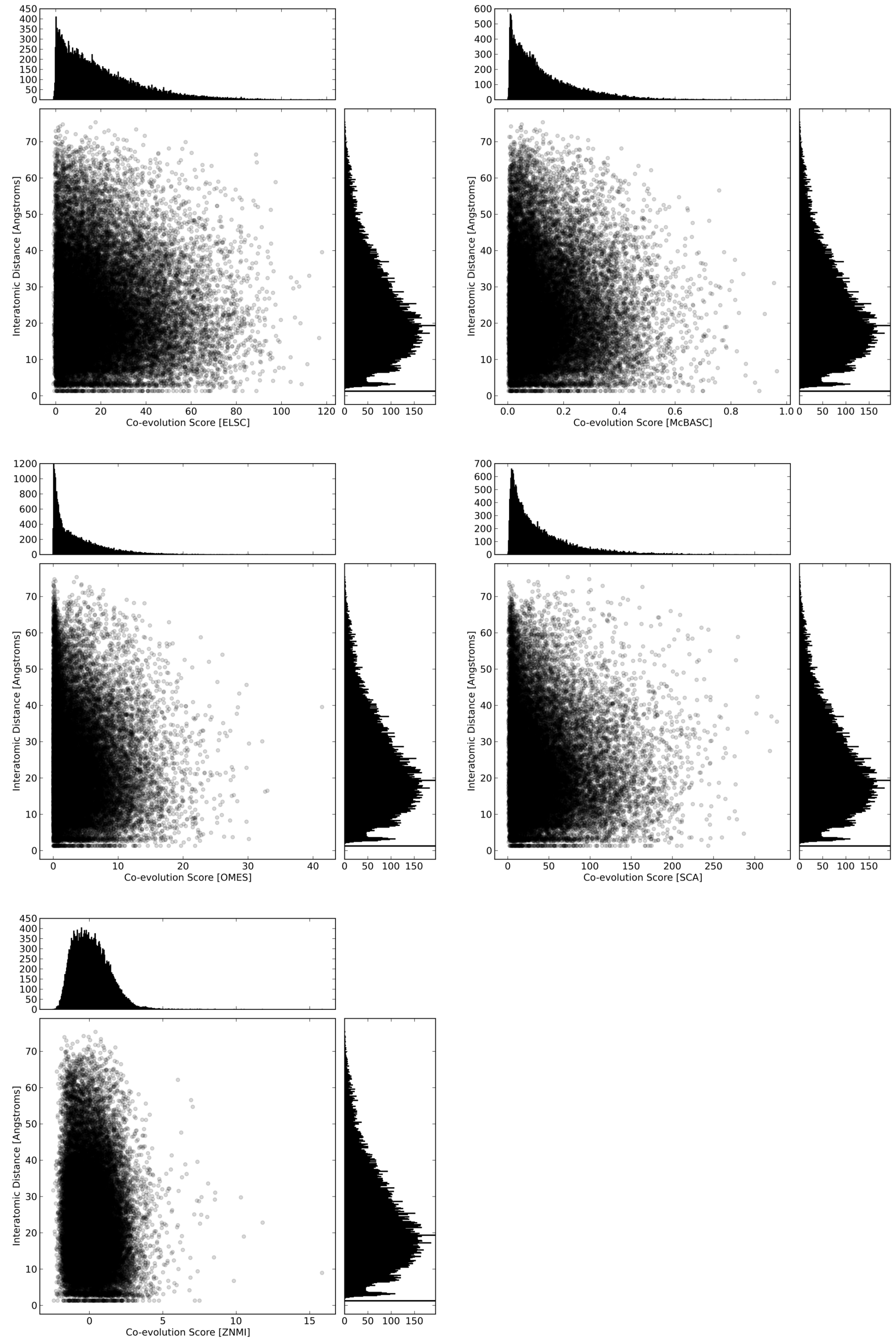

Figure S12: Lack of 3D structural proximity: CcpA

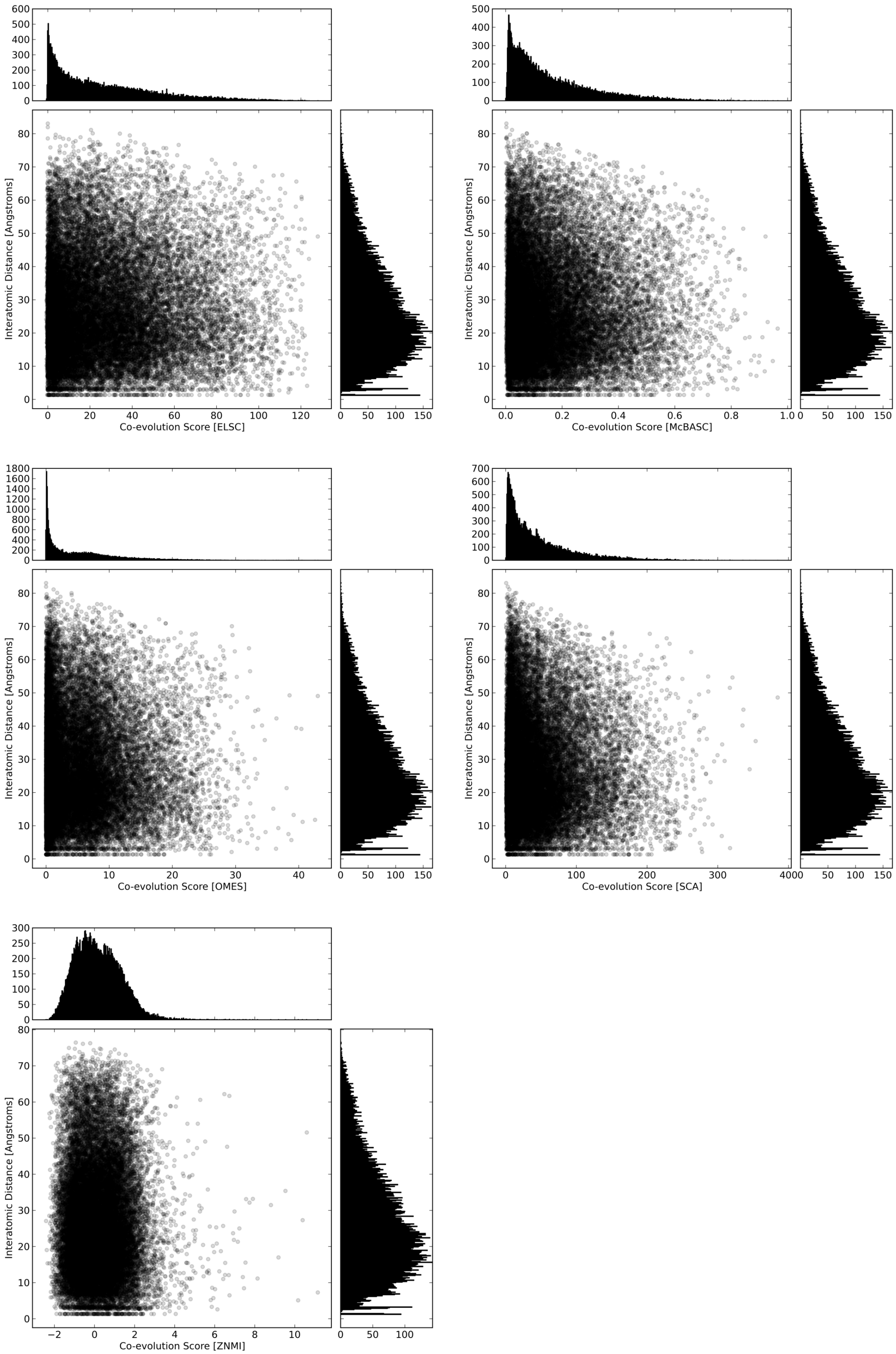

Figure S13: Lack of 3D structural proximity: GalRS

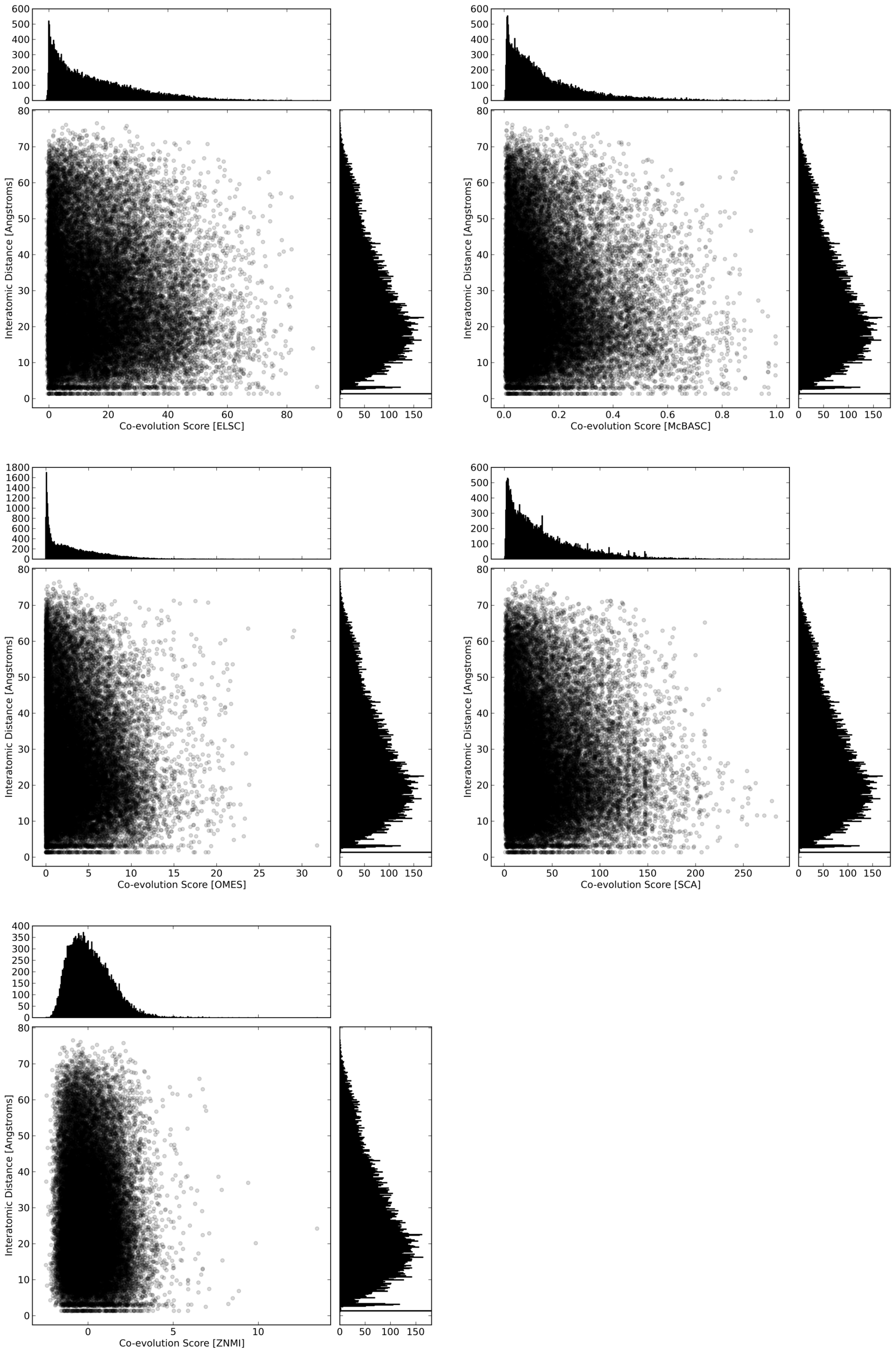

Figure S14: Lack of 3D structural proximity: GntR

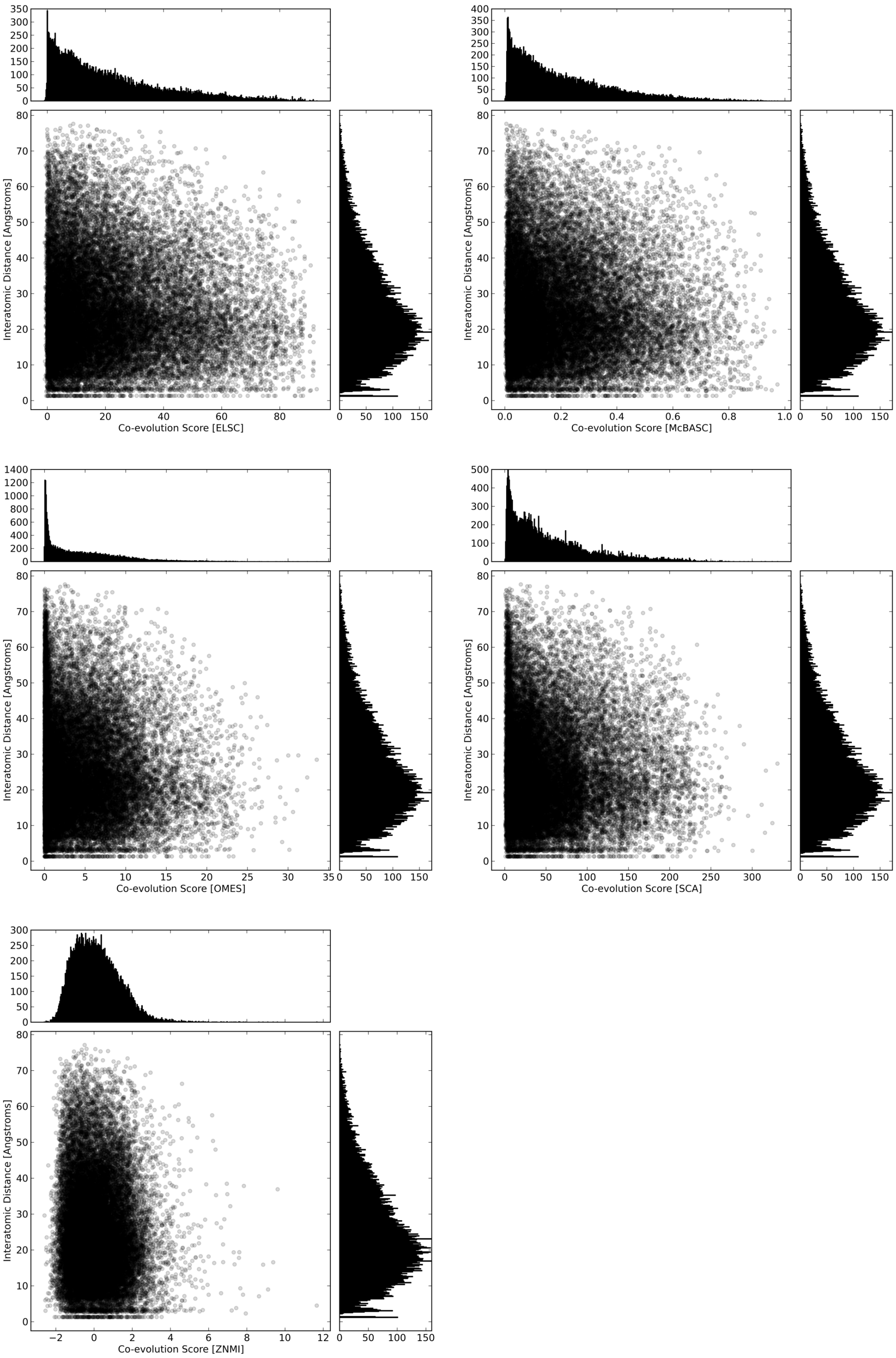

Figure S15: Lack of 3D structural proximity: PurR

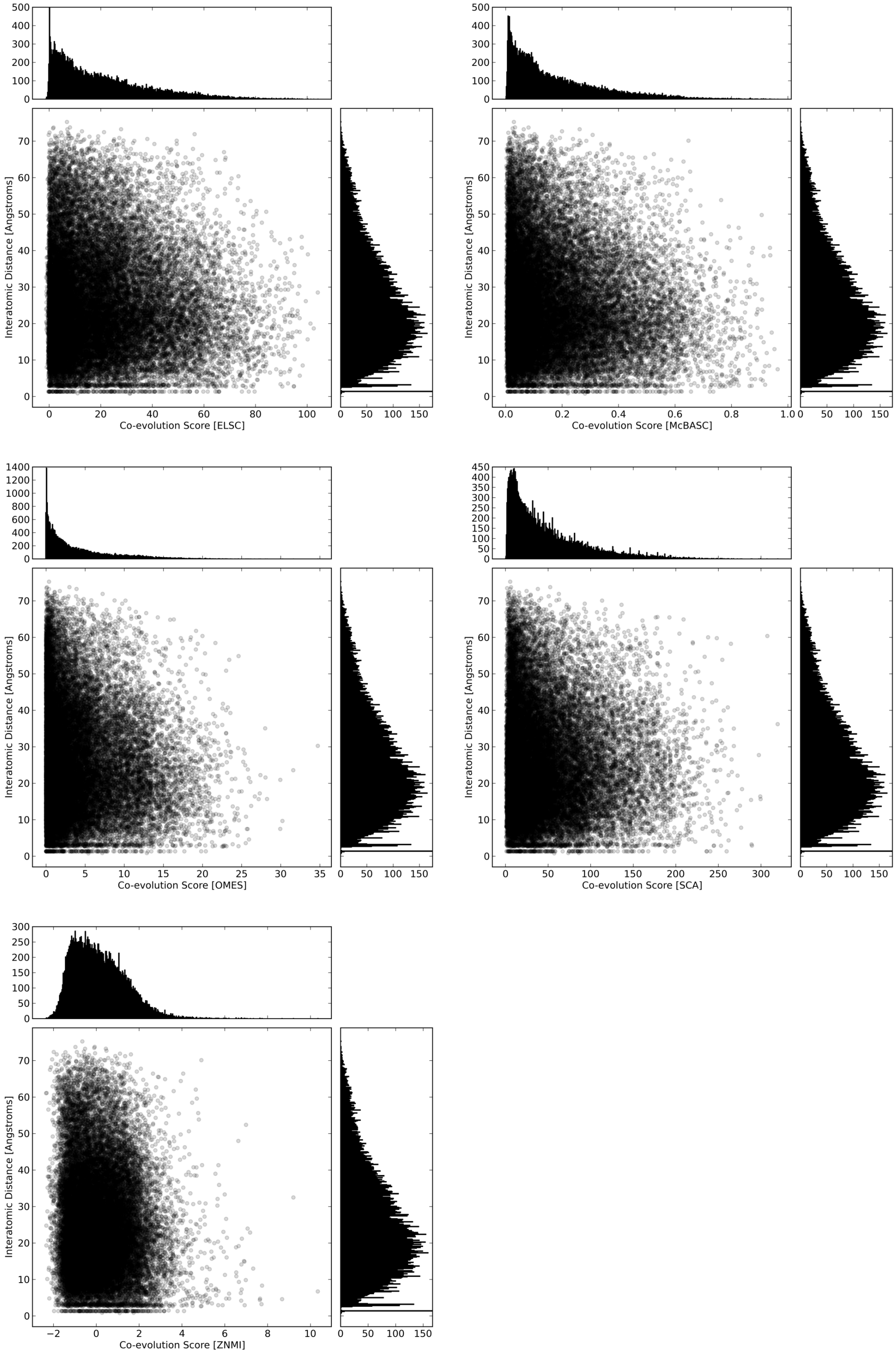

Figure S16: Lack of 3D structural proximity: RbsR-A

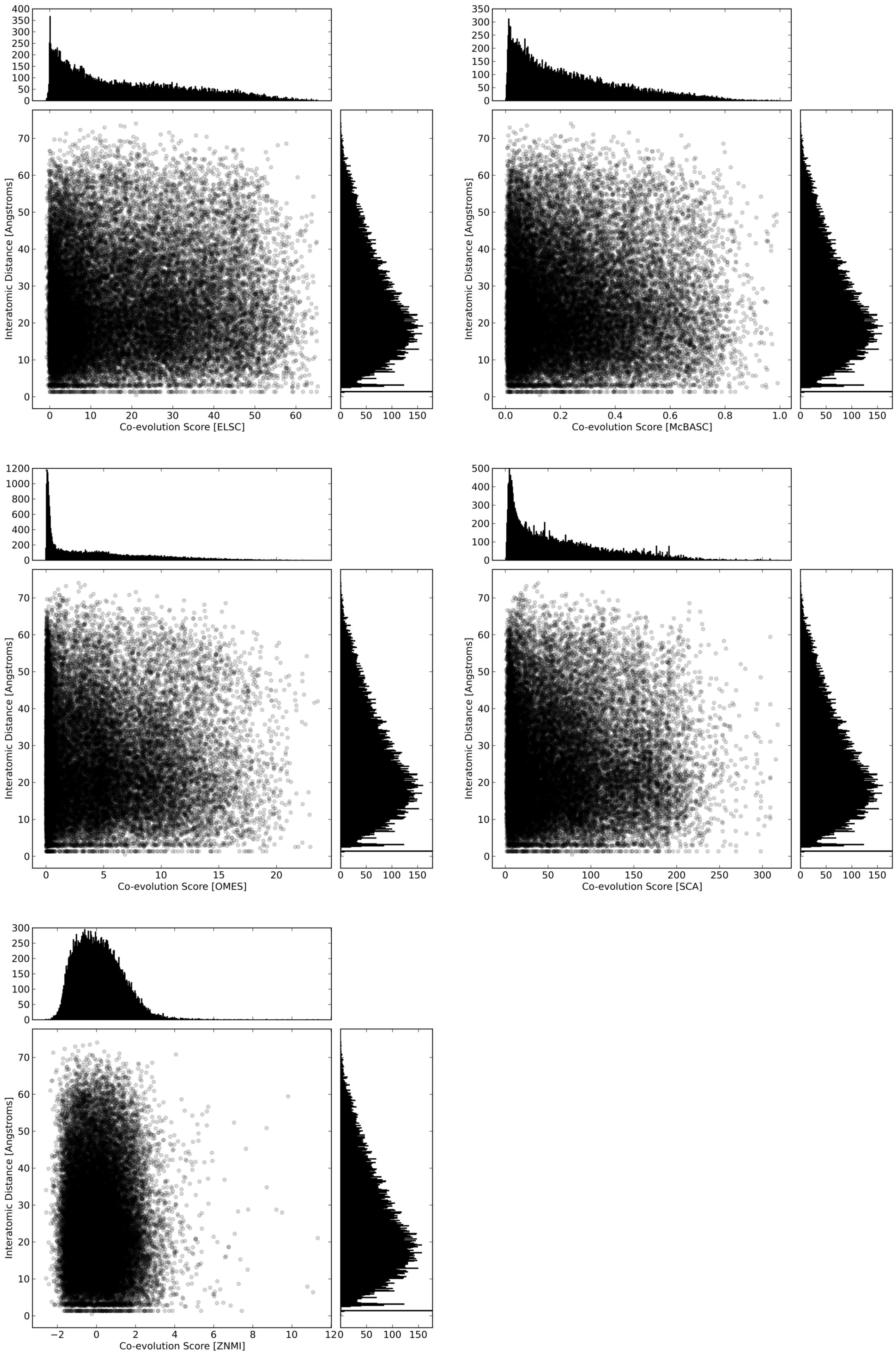

Figure S17: Lack of 3D structural proximity: TreR
