# Supplementary material for: Multiple Co-Evolutionary Networks Are Supported by the Common Tertiary Scaffold of the LacI/GalR Proteins
Source: PLoS One. 2013 Dec 31;8(12):e84398. doi: 10.1371/journal.pone.0084398 (PMC3877293; doi:10.1371/journal.pone.0084398)
Supplement: Data S4 — Nodal Jaccard analyses. Figures S18–S22. Nodal Jaccard analyses for all pairs of subfamilies. The Jaccard index for the set of N most highly-scoring positions (nodes), using each algorithm (figures), between all pairs of the six subfamilies (panels) is shown as a function of N (a-e, blue lines). The expected Jaccard index under the random model (black line, solid), 95% confidence interval of the expected index (red region), and maximum possible Jaccard index (black line, dotted) are shown. (PDF) [file pone.0084398.s004.pdf]

Supplemental data for:
Multiple co-evolutionary networks are supported by the common tertiary scaffold of the LacI/GalR proteins
File 4: Nodal Jaccard analyses

Daniel J. Parente and Liskin Swint-Kruse

List of Figures

S18 Nodal Jaccard analyses, all pairs of subfamilies: ELSC 3
S19 Nodal Jaccard analyses, all pairs of subfamilies: OMES 4
S20 Nodal Jaccard analyses, all pairs of subfamilies: McBASC 5
S21 Nodal Jaccard analyses, all pairs of subfamilies: SCA 6
S22 Nodal Jaccard analyses, all pairs of subfamilies: ZNMI 7

Figures S18-S22: Nodal Jaccard analyses for all pairs of subfamilies. The Jaccard index for the set of N most highly-scoring positions (nodes), using each algorithm (figures), between all pairs of the six subfamilies (panels) is shown as a function of N (a-e, blue lines). The expected Jaccard index under the random model (black line, solid), 95% confidence interval of the expected index (red region), and maximum possible Jaccard index (black line, dotted) are shown.

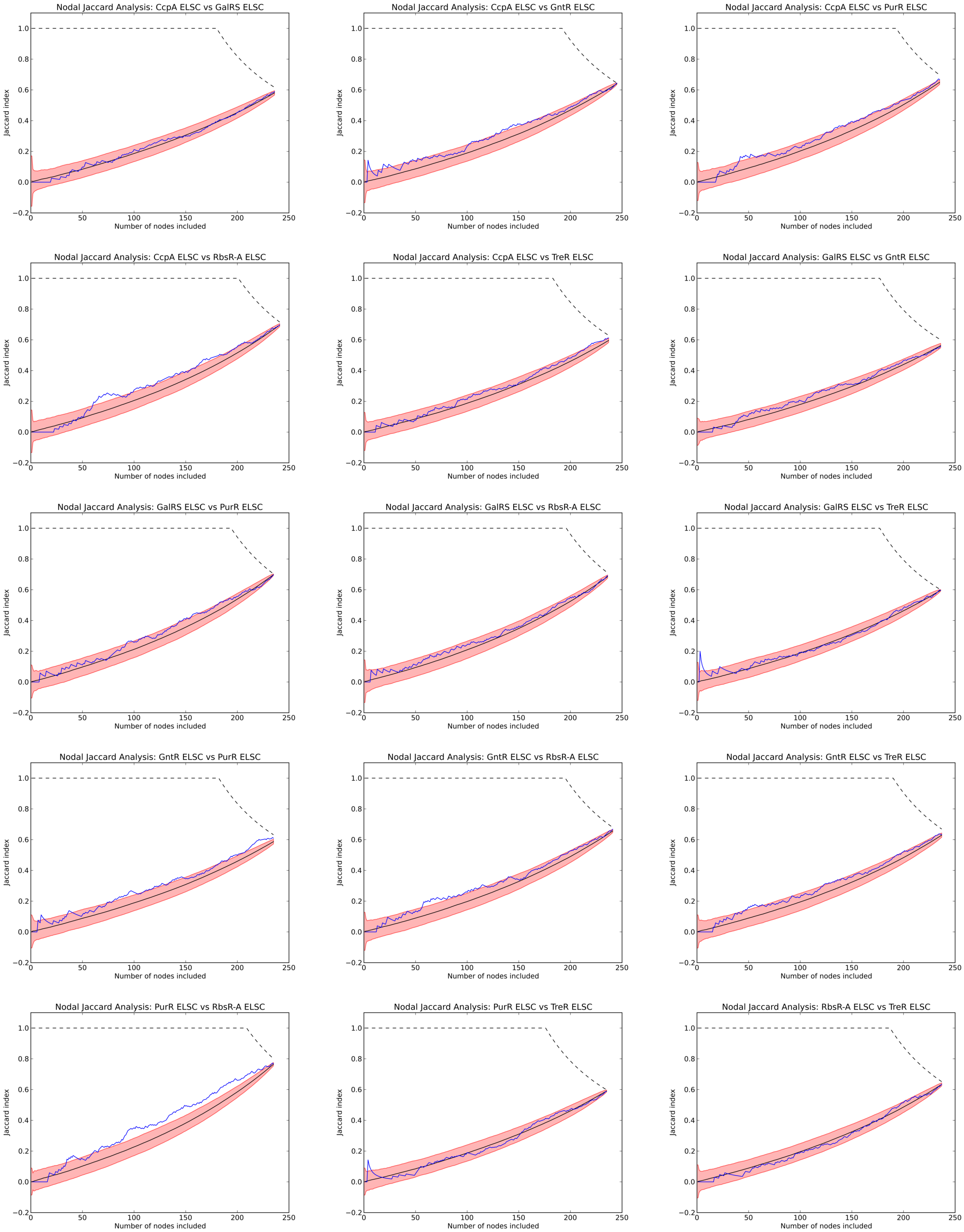

Figure S18: Nodal Jaccard analyses, all pairs of subfamilies: ELSC

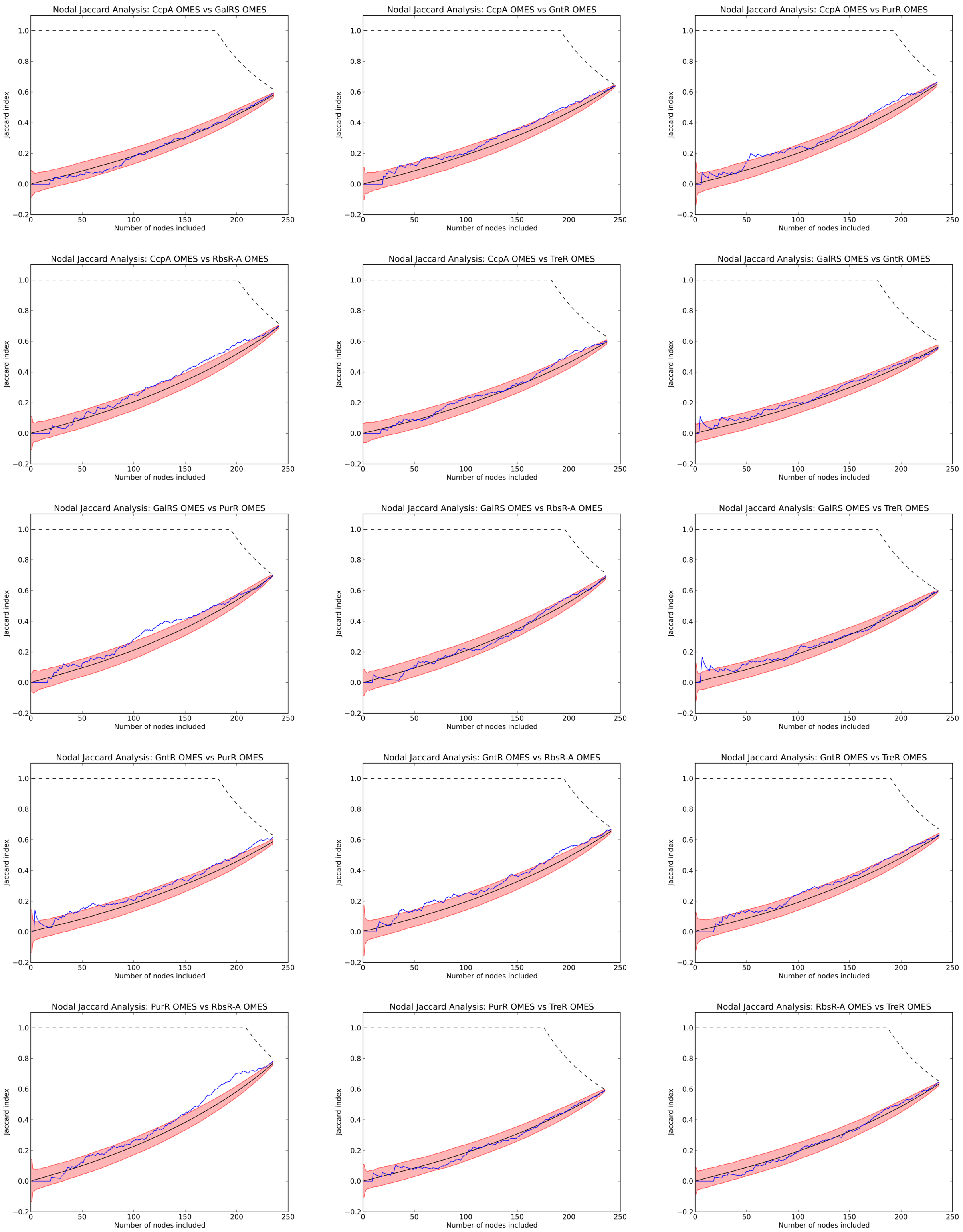

Figure S19: Nodal Jaccard analyses, all pairs of subfamilies: OMES

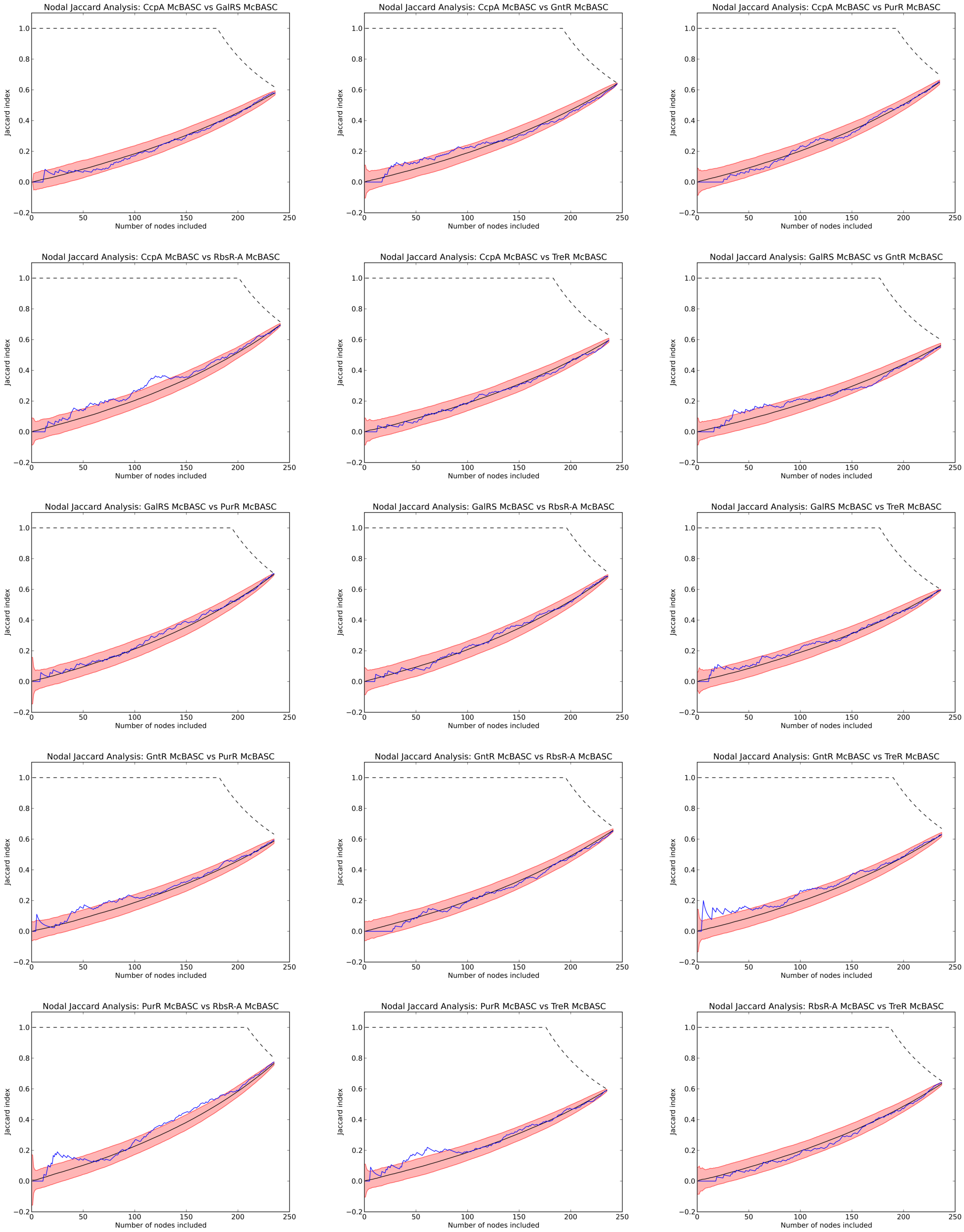

Figure S20: Nodal Jaccard analyses, all pairs of subfamilies: McBASC

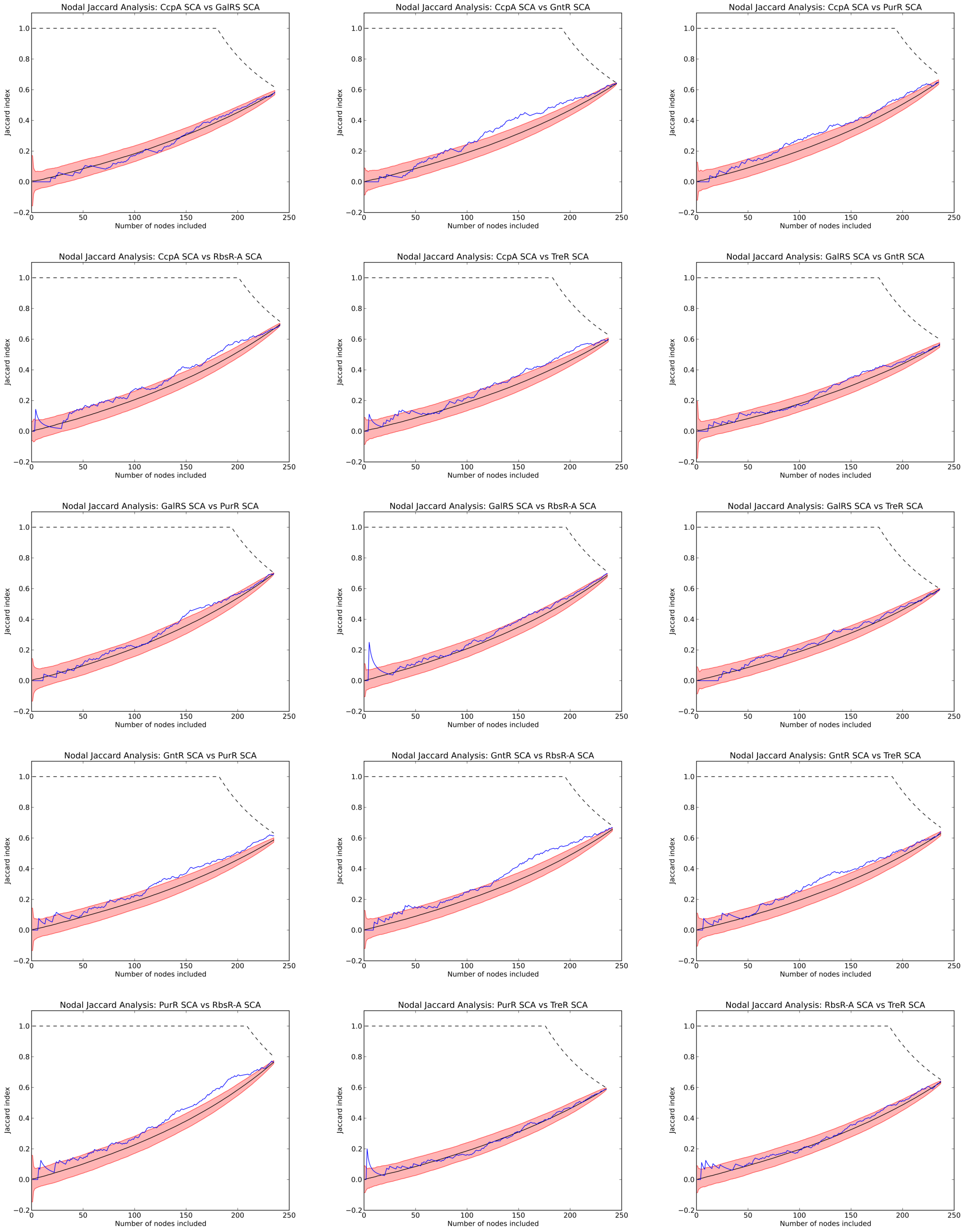

Figure S21: Nodal Jaccard analyses, all pairs of subfamilies: SCA

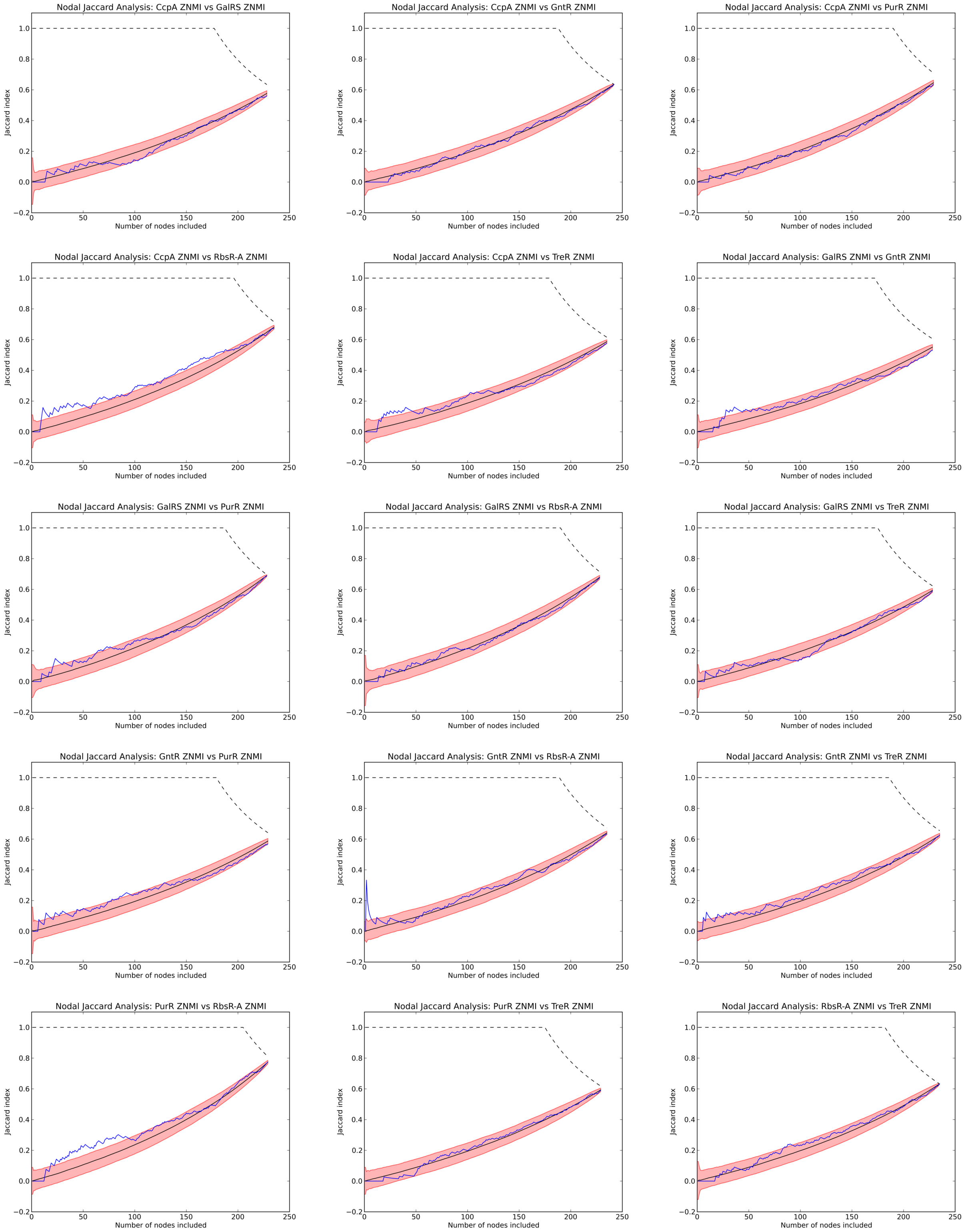

Figure S22: Nodal Jaccard analyses, all pairs of subfamilies: ZNMI
