# Supplementary material for: Multiple Co-Evolutionary Networks Are Supported by the Common Tertiary Scaffold of the LacI/GalR Proteins
Source: PLoS One. 2013 Dec 31;8(12):e84398. doi: 10.1371/journal.pone.0084398 (PMC3877293; doi:10.1371/journal.pone.0084398)
Supplement: Data S5 — GalR versus GalS and software pipeline. Figure S23. The GalR and GalS isorepressors: edgewise Jaccard analysis. The similarity of the set of N most highly co-evolving edges in the GalR and GalS isorepressors, as a function of the threshold (N) is shown (blue line). Comparison to the expected and 95% confidence interval of the random overlap model (black line and red region) and the perfect agreement model (black dotted line) are shown. Subpanels delineate comparisons made using different co-evolution analysis algorithms (ELSC, OMES, McBASC, SCA and ZNMI). Figure S24. The GalR and GalS isorepressors: nodal Jaccard analysis. The similarity of the set of N most highly co-evolving nodes in the GalR and GalS isorepressors, as a function of the threshold (N) is shown (blue line). Comparison to the expected and 95% confidence interval of the random overlap model (black line and red region) and the perfect agreement model (black dotted line) are shown. Subpanels delineate comparisons made using different co-evolution analysis algorithms (ELSC, OMES, McBASC, SCA and ZNMI). Figure S25. The GalR and GalS isorepressors: Highly co-evolving positions mapped to the structure. The 10 most strongly co-evolving positions in the GalR (spacefilled green) and GalS (spacefilled magenta) isorepressors are shown on the ITASSER model structure for GalR. Molecular graphics were created with UCSF Chimera. Figure S26. Analytical workflow, overview. Figure S27. Analytical workflow, ensemble-based co-evolution analysis. Table S1. Description of available programs. Table S2. Reference sequence alignment. (PDF) [file pone.0084398.s005.pdf]

Supplemental data for:
Multiple co-evolutionary networks are supported by the common tertiary scaffold of the LacI/GalR proteins
File 5: GalR versus GalS and software pipeline

Daniel J. Parente and Liskin Swint-Kruse

List of Figures

S23 The GalR and GalS isorepressors: edgewise Jaccard analysis 2
S24 The GalR and GalS isorepressors: nodal Jaccard analysis 3
S25 The GalR and GalS isorepressors: Highly co-evolving positions mapped to the structure 4
S26 Analytical workflow, overview 5
S27 Analytical workflow, ensemble-based co-evolution analysis 6

List of Tables

S1 Description of available programs 7
S2 Reference sequence alignment 9

Software

Software described in Figures S26-S27 and Table S1 is available on the web at `https://sourceforge.net/projects/coevolutils/`.

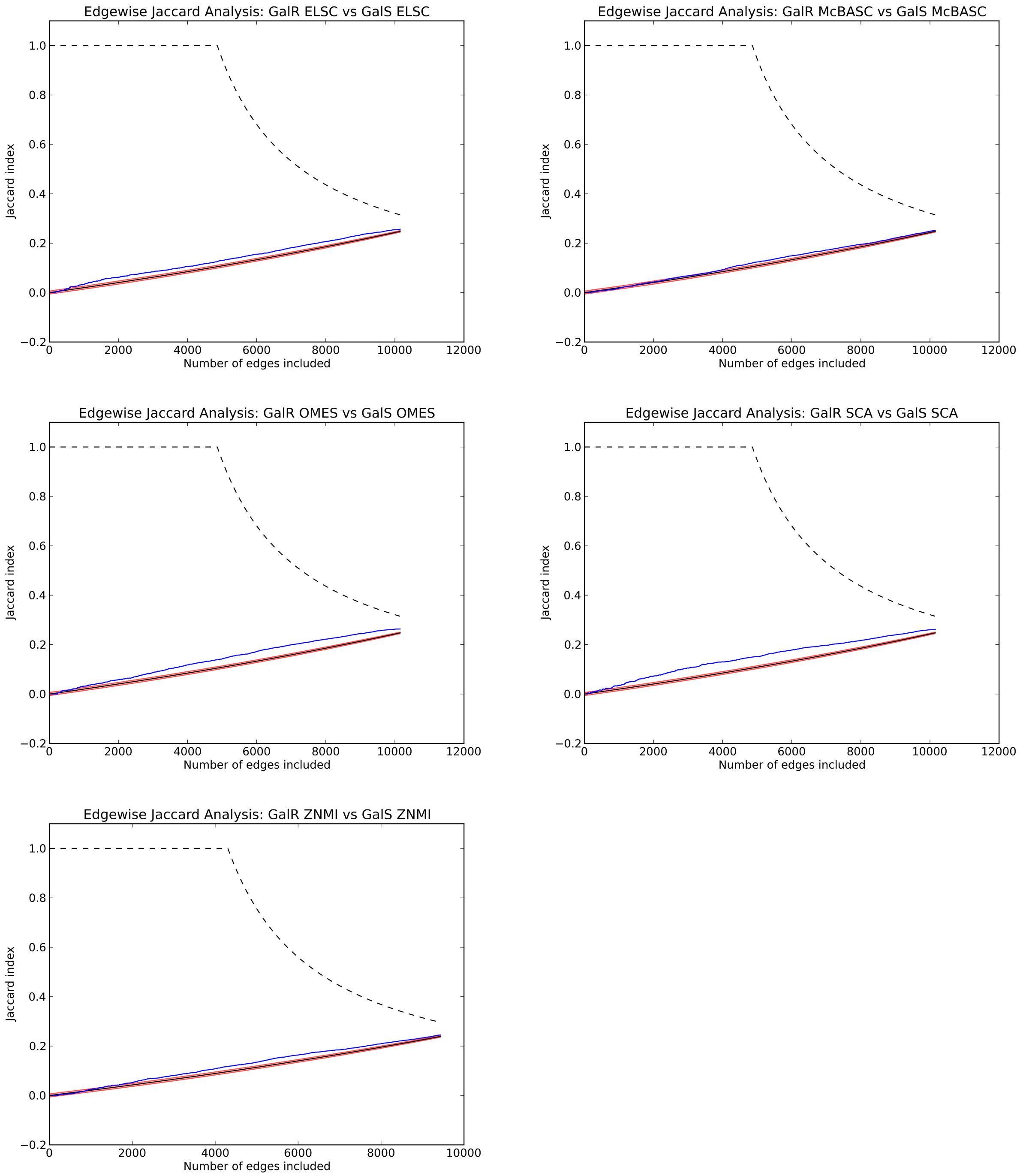

Figure S23: The GalR and GalS isorepressors: Edgewise Jaccard Analysis. The similarity of the set of N most highly co-evolving edges in the GalR and GalS isorepressors, as a function of the threshold (N) is shown (blue line). Comparison to the expected and 95% confidence interval of the random overlap model (black line and red region) and the perfect agreement model (black dotted line) are shown. Subpanels delineate comparisons made using different co-evolution analysis algorithms (ELSC, OMES, McBASC, SCA and ZNMI).

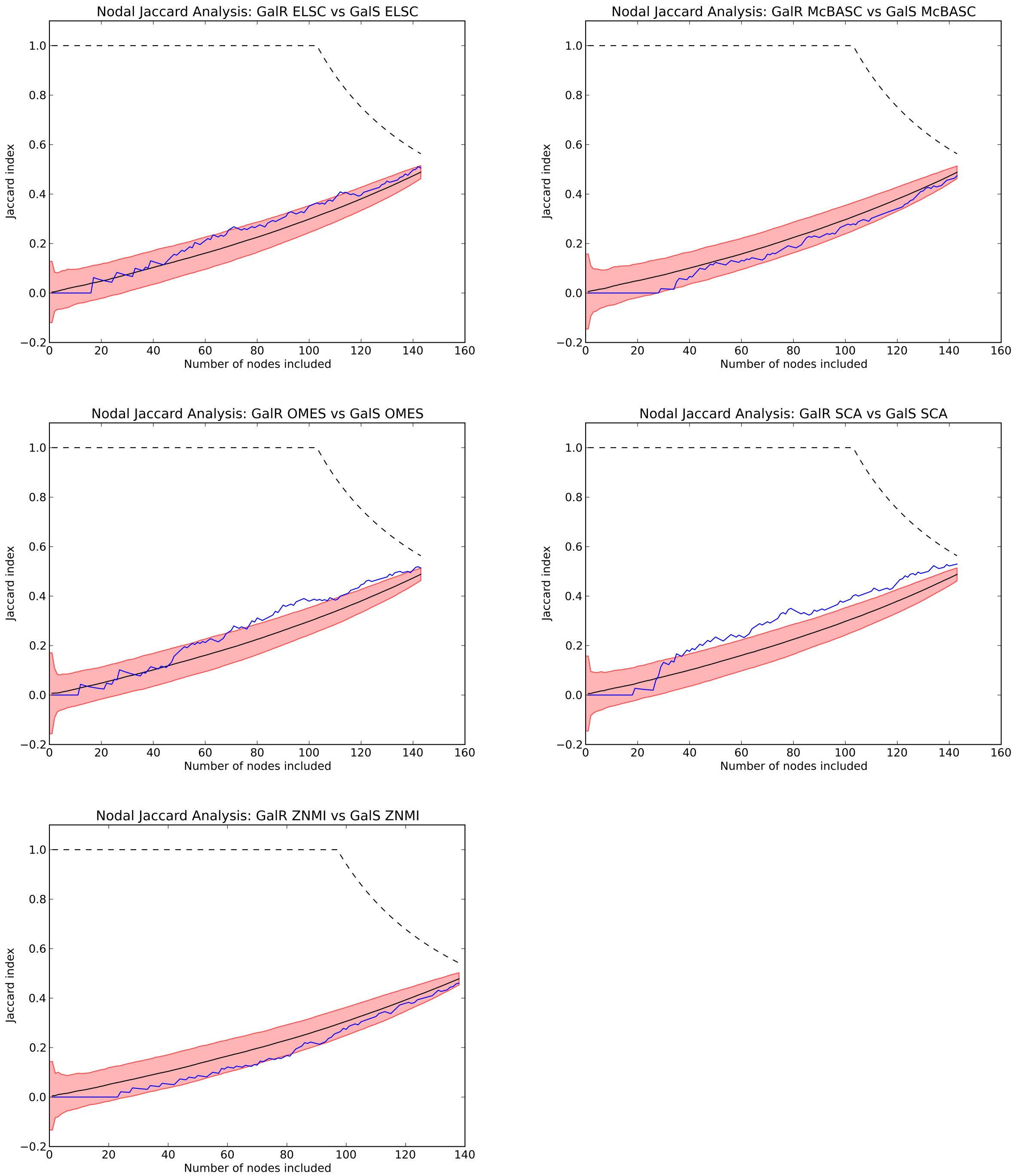

Figure S24: The GalR and GalS isorepressors: nodal Jaccard analysis. The similarity of the set of N most highly co-evolving nodes in the GalR and GalS isorepressors, as a function of the threshold (N) is shown (blue line). Comparison to the expected and 95% confidence interval of the random overlap model (black line and red region) and the perfect agreement model (black dotted line) are shown. Subpanels delineate comparisons made using different co-evolution analysis algorithms (ELSC, OMES, McBASC, SCA and ZNMI).

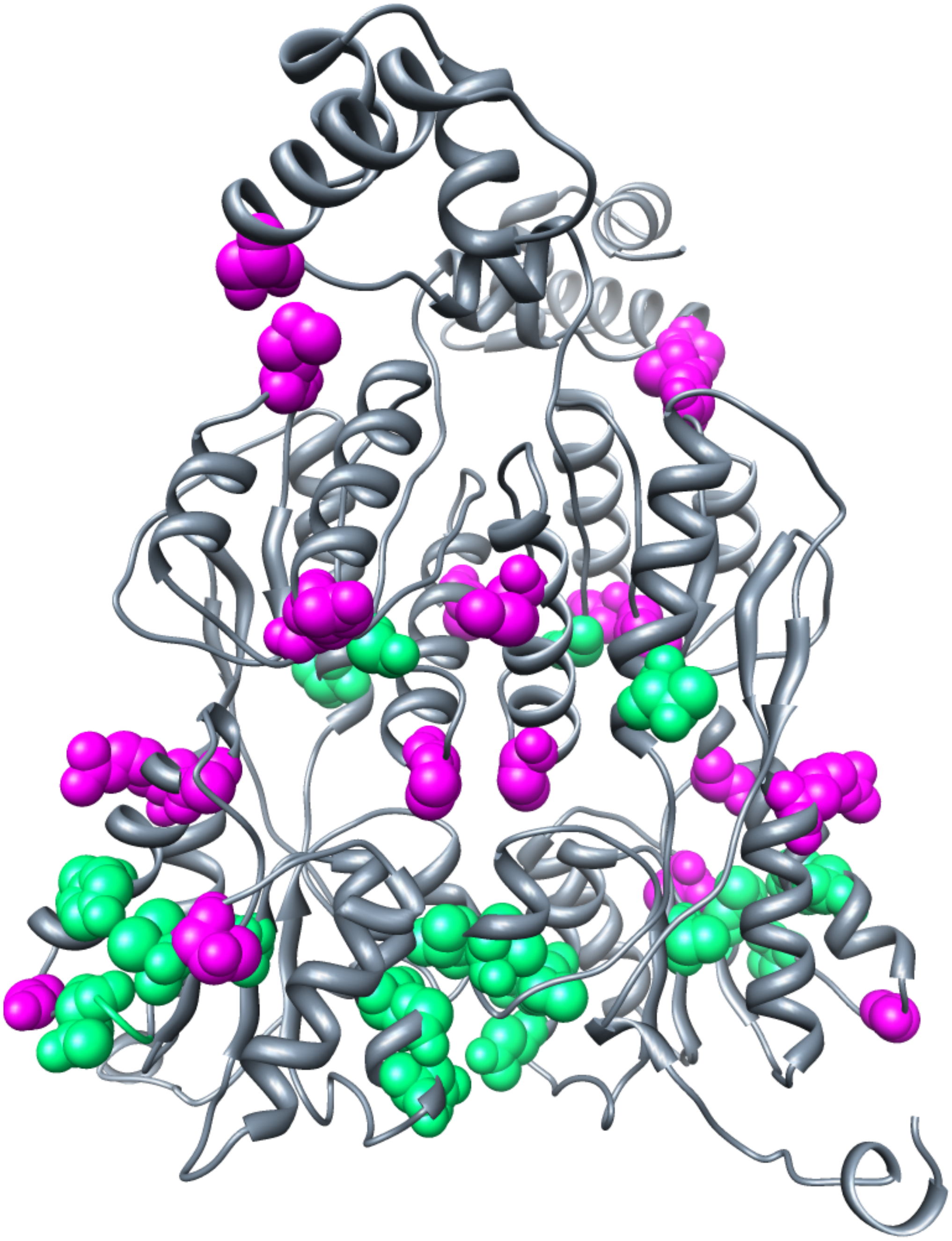

Figure S25: The GalR and GalS isorepressors: Highly co-evolving positions mapped to the structure. The 10 most strongly co-evolving positions in the GalR (spacefilled green) and GalS (spacefilled magenta) isorepressors are shown on the ITASSER model structure for GalR. Molecular graphics were created with UCSF Chimera.

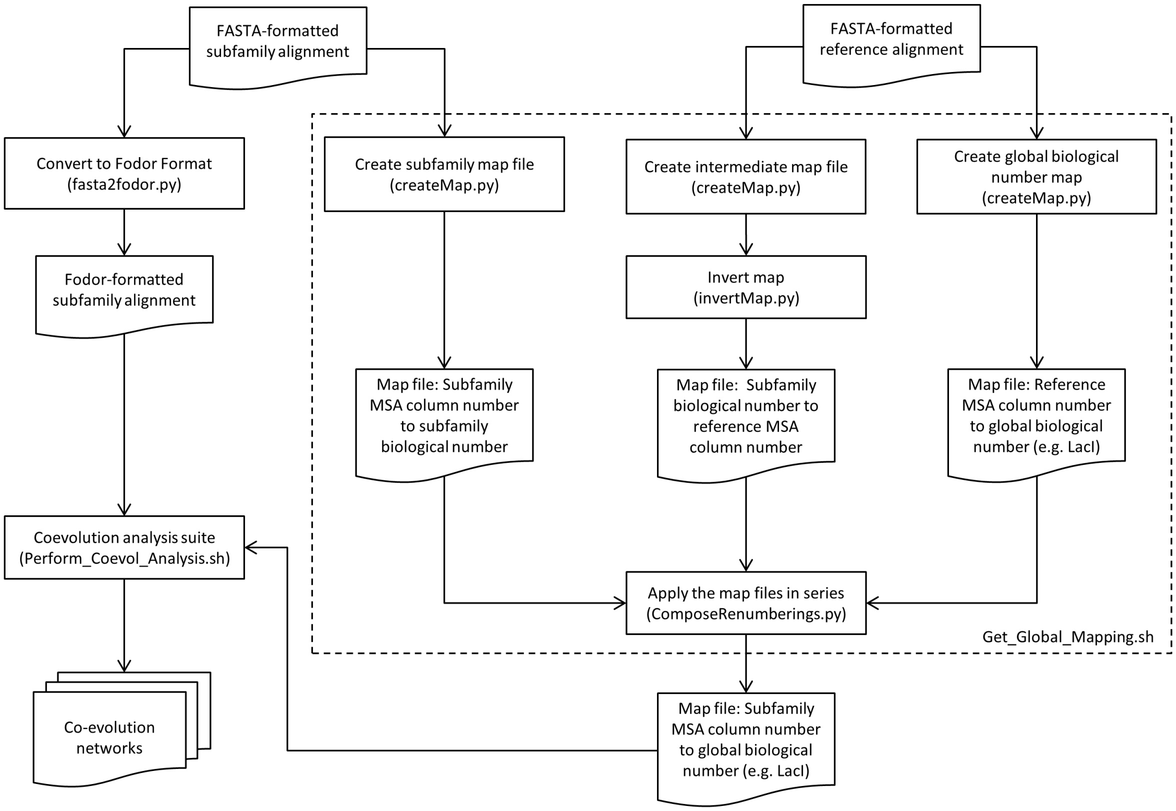

Figure S26: Analytical workflow, overview

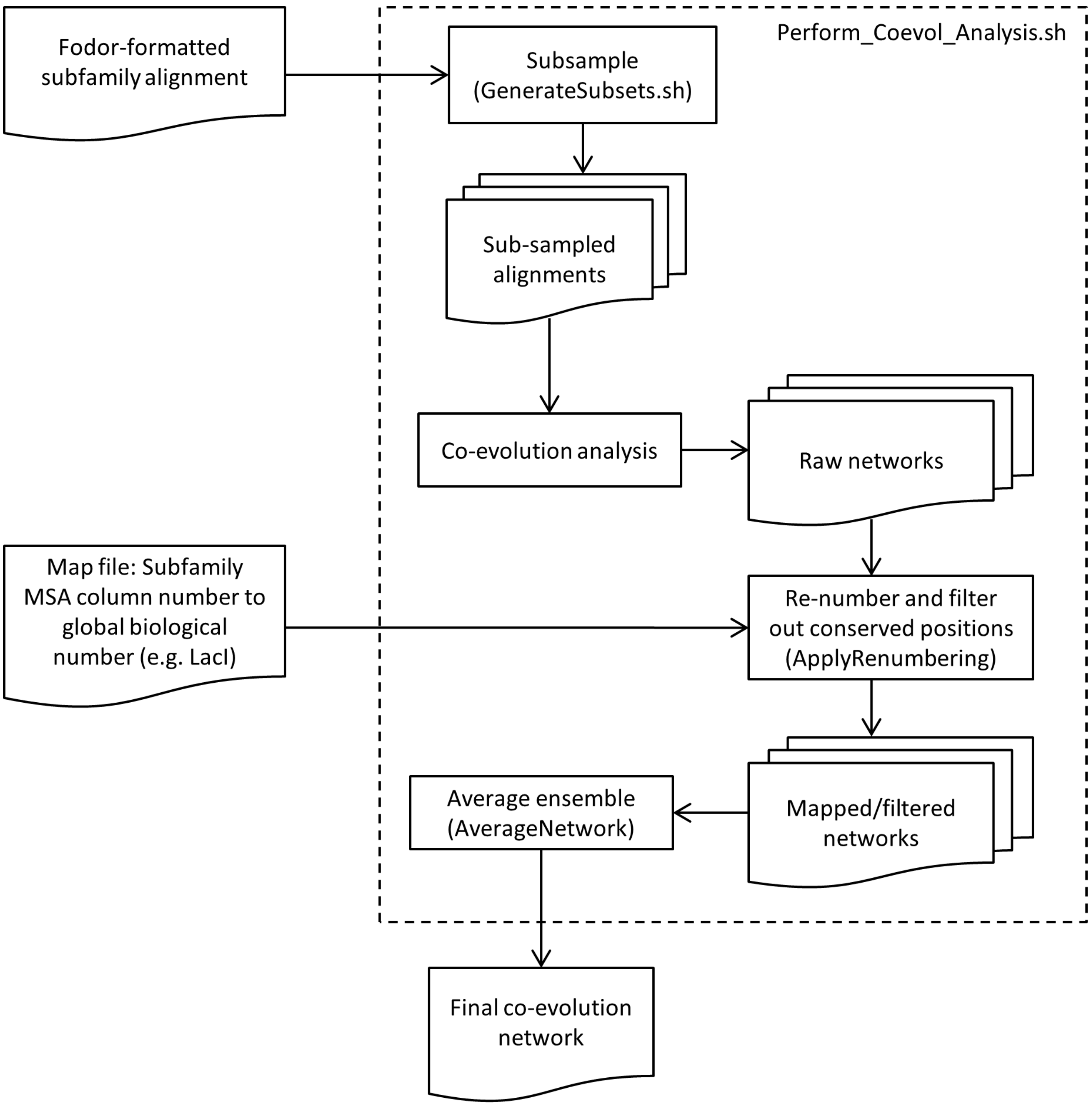

Figure S27: Analytical workflow, ensemble-based co-evolution analysis

Table S1: Description of available programs

Program	Language	Description
Perform_Coevol_Analysis.sh	Bash	Root-level script that performs a co-evolutionary analysis given an input Fodor-formatted file (i.e. an alignment compatible with A. Fodor's co-evolution package) and a mapping of all non-conserved positions to a common numbering system (e.g. LacI biological numbers).
≫ GenerateSubsets.sh	Bash	Subsidiary script of Perform_Coevol_Analysis that generates an ensemble of sub-sampled MSA containing a specified fraction of the total sequences (e.g. 90% or 50%).
≫ Mainscript.sh	Bash	Subsidiary script of Perform_Coevol_Analysis that (1) carries out co-evolution analyses for each algorithm, on every alignment in the ensemble, (2) filters out highly conserved positions, (3) maps the co-evolution network to a common (e.g. LacI) numbering system, and (4) Averages across the ensemble.
Get_Global_Mapping.sh	Bash	Calculates a table mapping the MSA column number of all non-conserved positions that are ungapped in the reference sequence to a common (e.g. LacI) numbering system.
≫ CreateMap.py	Python	Subsidiary script of Get_Global_Mapping.sh. Establishes a mapping between alignment number and biological number, for a given sequence in an alignment.
≫ ComposeRenumberings.py	Python	Subsidiary script of Get_Global_Mapping.sh. Applies several mappings (e.g. those generated by CreateMap.py) in series, to produce a new (composite) mapping directly linking the first and last map in the chain (i.e. without reference to intermediate mappings).
≫ InvertMap.py	Python	Subsidiary script of Get_Global_Mapping.sh. Inverts the directionality of a mapping (e.g. Column number $\rightarrow$ LacI number becomes LacI number $\rightarrow$ Column number).
ApplyRenumbering	C#	Transforms the sequence numbers of one column in a table, using a dictionary mapping (e.g. those created by Get_Global_Mapping.sh).
AverageNetwork	C#	Calculates the mean and standard deviation of the co-evolution scores produced for each alignment in a subsampled ensemble.
ColoredMSA	C#	Constructs a color-coded reference alignment figure, highlighting sites of conservation in each subfamily (similar to Figure 3), based on the sequence entropy of each position in the subfamily. Accepts a configuration file that specifies (1) the reference alignment, (2) the subfamily alignments, (3) the entropy-based threshold for "conserved" positions, (4) the layout geometry of the figure.
fodor2fasta.sh	Bash	Accepts a Fodor-formatted file and converts it to FASTA format.
fasta2fodor.py	Python	Accepts a FASTA-formatted file and converts it to Fodor format.
ZNMI	C#	Re-implementation of the ZNMI algorithm in C#.
AlignMultiNetworks.py	Python	Accepts several co-evolution networks and collates a table of scores for each edge, showing its score in each input co-evolution network.

Continued on next page

Table S1 – *Continued from previous page*

Program	Language	Description
CompileMasterTable	C#	Accepts tables associating nodes with analysis parameters and collates a table of scores for each node, showing its score in each of the input tables.
OrderEdges.py	Python	Rank-orders the edges of a co-evolution network.
OrderNodes.py	Python	Rank-orders the nodes of a co-evolution network, based on the score of its strongest edge.
Jaccard.py	Python	Calculates the Jaccard index for a pair of rank-ordered positions/edges, for all possible threshold values, and compares to the "random" model. Outputs a table suitable for use with PlotJaccardEdge.py or PlotJaccardNode.py for final figure construction.
PlotJaccardEdge.py	Python	Constructs a plot of the Jaccard index for all threshold values, using the output of Jaccard.py, labeling the axis for edges.
PlotJaccardNode.py	Python	Constructs a plot of the Jaccard index for all threshold values, using the output of Jaccard.py, labeling the axis for nodes.
ExtractDistanceNetwork.py	Python	Extracts a distance network in weighted edgelist format for all pairs of ATOM records in the specified chains of a PDB.
MeasureScoreVsDistance.py	Python	Uses a distance and co-evolution network to construct a table associating physical distance with co-evolution score.
CliContact.py	Python	Detects contacts between a set of protein and ligand ATOM/HETATM records (i.e. compatible with PDB format), implementing the RESMAP contact criteria.
FilterFASTABySequence.py	Python	Filters a FASTA-formatted sequence alignment, removing all sequences not contained in an inclusion list.
ExtractNodeData.py	Python	Parses a co-evolution network and reports all scores associated with a given node.
GetPovrayEdgeDesc.py	Python	Writes a PovRay scene descriptor file to display key edges on to a dimeric backbone trace. This script is LacI/GalR specific, but easier modifiable for use with other systems.
IdentifyBasisVectors	C#	Identifies a set of disparate (representative) sequences from a subfamily, using an iterative dynamic programming approach. These can be used as input seed sequences to BLAST.

Table S2: Reference sequence alignment

Subfamily	Aligned Sequence
CcpA	--MSNITIYDVAREANVSMATVSRVVNGNPNVKPTTRKKVLEAIERLGYRPNAVARGLAS<br>KKTTTVGVIIPDISSIFYSELARGIEDIATMYKYNIILSNSD-QNMEKELHLLNTMLGKQ<br>VDGIVFMGGNITDEHVAEFKRS-PVPIV-LAASVEEQEE-TPSVAIDYEQAIYDAVKLLV<br>DKGHTDIAFVSGPMAEPINRSKKLQGYKRALEEANLPFNEQ-FVAEGDYTYDSGLEALQH<br>LMSLDKK--PTAILSATDEMALGIIHAAQDQGLSIPEDLDIIGFDNTRLSLMVRPQLSTV<br>VQPTYDIGAVAMRLLTKLMNKEPVEEHIVELPHRIELRKSTKS-----------------<br>------------
GalR	----MATIKDVARLAGVSVATVSRVINNSPKASEASRLAVHSAMESLSYHPNANARALAQ<br>QTTETVGLVVGDVSDPFFGAMVKAVEQVAYHTGNFLLIGNGY-HNEQKERQAIEQLIRHR<br>CAALVVHAKMIPDADLASLMKQM-PGMV-LINRILPGFE-NRCIALDDRYGAWLATRHLI<br>QQGHTRIGYLCSNHSISDAE-DRLQGYYDALAESGIAANDR-LVTFGEPDESGGEQAMTE<br>LLGRGRN--FTAVACYNDSMAAGAMGVLNDNGIDVPGEISLIGFDDVLVSRYVRPRLTTV<br>RYPIVTMATQAAELALALADNRPLPEITNVFSPTLVRRHSVSTPSLEASHHATSD-----<br>------------
GalS	----MITIRDVARQAGVSVATVSRVLNNSTLVSADTREAVMKAVSELDYRPNANAQALAT<br>QVSDTIGVVVMDVSDAFFGALVKAVDLVAQQHQKYVLIGNSY-HEAEKERHAIEVLIRQR<br>CNALIVHSKALSDDELAQFMDNI-PGMV-LINRVVPGYA-HRCVCLDNLSGARMATRMLL<br>NNGHQRIGYLSSSHGIEDDA-MRKAGWMSALKEQDIIPPES-WIGAGTPDMPGGEAAMVE<br>LLGRNLQ--LTAVFAYNDNMAAGALTALKDNGIAIPLHLSIIGFDDIPIARYTDPQLTTV<br>RYPIASMAKLATELALQGAAGNIDPRASHCFMPTLVRRHSVATRQNAAAITNSTNQAM--<br>------------
GntR	MKKKRPVLQDVADRVGVTKMTVSRFLRNPEQVSVALRGKIAAALDELGYIPNRAPDILSN<br>ATSRAIGVLLPSLTNQVFAEVLRGIESVTDAHGYQTMLAHYG-YKPEMEQERLESMLSWN<br>IDGLILTERTH-TPRTLKMIEVAGIPVVELMDSKSPC-L-DIAVGFDNFEAARQMTTAII<br>ARGHRHIAYLGARL-DERTI-IKQKGYEQAMLDAGLVPY--SVMVEQSSSYSSGIELIRQ<br>ARRE-YPQ-LDGVFCTNDDLAVGAAFECQRLGLKVPDDMAIAGFHGHDIGQVMEPRLASV<br>LTPRERMGSIGAERLLARIRGESVTPKMLDLGFTLSPGGSI-------------------<br>------------
LacI	--MKPVTLYDVAEYAGVSYQTVSRVVNQASHVSAKTREKVEAAMAELNYIPNRVAQQLAG<br>KQSLLIGVATSSLALHAPSQIVAAIKSRADQLGASVVVSMVERSGVEACKAAVHNLLAQR<br>VSGLIINYPLDDQDAIAVEAACTNVPAL-FLDVSDQTP--INSIIFSHEDGTRLGVEHLV<br>ALGHQQIALLAGPLSSVSAR-LRLAGWHKYLTRNQIQPI---AEREGDWSAMSGFQQTMQ<br>MLNEGIV--PTAMLVANDQMALGAMRAITESGLRVGADISVVGYDDTEDSSCYIPPLTTI<br>KQDFRLLGQTSVDRLLQLSQGQAV-KGNQLLPVSLVKRKTTLAPNTQTASPRALADSLMQ<br>LARQVSRLESGQ
PurR	----MATIKDVAKRANVSTTTVSHVINKTRFVAEETRNAVWAAIKELHYSPSAVARSLKV<br>NHTKSIGLLATSSEAAYFAEIIEAVEKNCFQKGYTLILGNAW-NNLEKQRAYLSMMAQKR<br>VDGLLVMCSEYPEPLLAMLEEYRHIPMV-VMDWGEAKADFTDAVIDNAFEGGYMAGRYLI<br>ERGHREIGVIPGPLERNTGA-GRLAGFMKAMEEAMIKVPES-WIVQGDFEPESGYRAMQQ<br>ILSQPHR--PTAVFCGGDIMAMGALCAADEMGLRVPQDVSLIGYDNVRNARYFTPALTTI<br>HQPKDSLGETAFNMLLDRIVNKREEPQSIEVHPRLIERRSVADGPFRDYRR---------<br>------------
RbsR-A	----MATMKDVARLAGVSTSTVSHVINKDRFVSEAITAKVEAAIKELNYAPSALARSLKL<br>NQTHTIGMLITASTNPFYSELVRGVERSCFERGYSLVLCNTE-GDEQRMNRNLETLMQKR<br>VDGLLLLCTETHQPSREIMQRYPTVPTV-MMDWAPFDGD-SDLIQDNSLLGGDLATQYLI<br>DKGHTRIACITGPLDKTPAR-LRLEGYRAAMKRAGLNIPDG-YEVTGDFEFNGGFDAMRQ<br>LLSHPLR--PQAVFTGNDAMAVGVYQALYQAELQVPQDIAVIGYDDIELASFMTPPLTTI<br>HQPKDELGELAIDVLIHRITQPTLQQQRLQLTPILMERGSA-------------------<br>------------

Continued on next page

Table S2 – *Continued from previous page*

Subfamily	Aligned Sequence
TreR	-MQNRLTIKDIARLSGVGKSTVSRVLNNESGVSQLTRERVEAVMNQHGFSPSRSARAMRG<br>QSDKVVAIIVTRLDSLSENLAVQTMLPAFYEQGYDPIMMESQ-FSPQLVAEHLGVLKRRN<br>IDGVVLFGFTG--ITEEMLAHWQSS-LV-LLARD--AKG-FASVCYDDEGAIKILMQRLY<br>DQGHRNISYLGVPHSDVTTGKRRHEAYLAFCKAHKLHPV----AALPGLAMKQGYENVAK<br>VI----TPETTALLCATDTLALGASKYLQEQRID---TLQLASVGNTPLMKFLHPEIVTV<br>DPGYAEAGRQAACQLIAQVTGRSE-PQQIIIPATLS------------------------<br>------------
